# The cytoplasmic-nuclear transport of DDX3X promotes immune-mediated liver injury in mice regulated by endoplasmic reticulum stress

**DOI:** 10.1038/s41419-024-07076-9

**Published:** 2024-09-30

**Authors:** Zihao Fan, Ling Xu, Yao Gao, Yaling Cao, Yuan Tian, Zhenzhen Pan, Linlin Wei, Sisi Chen, Xiangying Zhang, Mei Liu, Feng Ren

**Affiliations:** 1 Beijing Institute of Hepatology/Beijing Youan Hospital, Capital Medical University, 100069 Beijing, China; 2 Present Address: Department of Oncology, Beijing Youan Hospital, Capital Medical University, 100069 Beijing, China

**Keywords:** Liver diseases, Experimental models of disease, Mechanisms of disease, Immunopathogenesis

## Abstract

Immune-mediated liver injury is a common characteristic of various liver diseases, including autoimmune and viral hepatitis. Here, we investigated the role of DEAD-box helicase 3, X-linked (DDX3X) in immune-mediated liver injury. Liver injury was induced in C57BL/6J mice via concanavalin A (Con A). DDX3X hepatocyte-specific knockout (DDX3X^ΔHep^) mice and control (DDX3X^fl/fl^) mice were utilized to investigate the role of DDX3X in liver injury. Primary hepatocytes were treated with tunicamycin (TM) to induce ER stress in vitro. The expression of DDX3X in patients with various liver diseases was evaluated. Hepatic DDX3X expression increased, and DDX3X translocated from the cytoplasm to the nucleus during Con A-induced liver injury. DDX3X deficiency ameliorated mouse liver injury and reduced ER stress in liver tissue. The inhibition of ER stress with 4-PBA significantly attenuated liver injury while decreasing DDX3X levels in liver tissue. However, the upregulation of hepatic DDX3X expression reversed Con A-induced liver injury and negated the protective effect of 4-PBA. Mechanistically, the nuclear translocation of DDX3X promoted ER stress-induced apoptosis through the transcriptional induction of CHOP. Moreover, DDX3X was elevated and translocated into the nucleus in patients with HBV-LF and AIH. Additionally, serum DDX3X levels markedly increased in patients with HBV-LF, and a consistent decrease in DDX3X was associated with a good prognosis. The cytoplasmic-to-nuclear translocation of DDX3X promotes ER stress-induced apoptosis, which is an obligatory step that drives hepatic necrosis and tissue damage. Notably, DDX3X is a potential therapeutic target for immune-mediated liver injury.

## Introduction

Liver failure (LF) is a clinical syndrome resulting from hepatocellular apoptosis and hemorrhagic necrosis that can be triggered by hepatitis B virus (HBV) infection, autoimmune hepatitis, or other etiologies. While numerous studies have established that immune-mediated hepatic injury plays a central role in this serious and progressive disease [1, 2], the intrinsic mechanisms underlying LF disease progression remain incompletely understood.

Concanavalin A (Con A), a lectin derived from jack bean plants, is commonly used to induce immune-mediated hepatic injury in murine models [3]. Therefore, the well-established experimental model induced by Con A provides a valuable opportunity to explore the molecular mechanisms underlying immune-mediated liver injury. Previous studies have shown that Con A-induced immune-mediated hepatic injury may trigger endoplasmic reticulum stress (ER stress) and activate the unfolded protein response (UPR) [4]. The UPR alleviates ER stress by regulating related proteins such as GRP78 and CHOP. However, persistent ER stress can lead to a significant increase in CHOP expression, initiating apoptosis. CHOP may further promote cell death by enhancing apoptosis and the expression of inflammatory factors, or by modulating oxidative stress responses. Nevertheless, the mechanisms by which the CHOP pathway promotes hepatocyte apoptosis in Con A-induced liver injury remain unclear.

DEAD-box helicase 3, X-linked (DDX3X), a member of the DEAD (Asp-Glu-Ala-Asp) box helicase family, is implicated in nearly all stages of RNA metabolism, including transcription, pre-mRNA splicing, RNA export, and translation [5]. DDX3X is expressed ubiquitously in human tissues and participates in various biological processes; it is localized in the cytoplasm and nucleus and performs distinct functions, such as translation and transcription processes. Dysregulation of its subcellular location could contribute to multiple diseases [6, 7]. The physiological functions of DDX3X, including assisting in viral replication, tumorigenesis, activation of innate immunity, and cell cycle regulation, have been extensively studied [8–11]. Studies have determined that DDX3X is a common component of stress granules (SGs) and the NLRP3 inflammasome, acting as a rheostat-like mechanism for regulating live-or-dead cell fate decisions under stress conditions [12]. Recent studies have determined that hepatocyte DDX3X protects against drug-induced acute liver injury by controlling stress granule formation and oxidative stress [13]. However, whether DDX3X contributes to immune-mediated liver injury remains unclear.

The aim of this study was to investigate the role of DDX3X in immune-mediated liver injury. We hypothesize that DDX3X promotes immune-mediated liver injury in mice by modulating endoplasmic reticulum stress (ER stress)-induced hepatocyte apoptosis. Our data revealed the following: (1) DDX3X translocates from the cytoplasm to the nucleus during Con A-induced liver injury; (2) DDX3X deficiency protects mice from Con A-induced liver injury by regulating ER stress; (3) the nuclear translocation of DDX3X transcriptionally induces CHOP under ER stress; and (4) DDX3X translocates into the nucleus in the liver tissues of HBV-LF patients and AIH patients. Therefore, our findings highlight that DDX3X may serve as a potential therapeutic target for immune-mediated liver injury.

## Materials and methods

### Patients

To determine the expression of DDX3X in the development and progression of HBV-LF, serum samples were collected from 60 patients with CHB, 60 patients with HBV-LF, and 30 healthy controls. The clinical characteristics and details of the subjects are summarized in Supplementary Table 1. We collected normal liver samples from 8 donor livers for liver transplantation, 18 patients with CHB who underwent liver puncture biopsy, 18 autoimmune hepatitis (AIH) who underwent liver puncture biopsy, 18 HBV-LF liver, samples from patients who underwent liver transplantation. (approval number: 2019-989).

In this study, we excluded patients with hepatitis A, C, D, or E and those with Epstein–Barr virus, cytomegalovirus, or human immunodeficiency virus. The study was approved by the medical ethics committee of Beijing Youan Hospital, Capital Medical University, and written informed consent was obtained from each patient. The procedures followed were performed in accordance with the ethical standards of the responsible committee on human experimentation and with the Helsinki Declaration of 1975, as revised in 1983. All experiments were performed in a double-blind manner.

### Animals

Male C57BL/6J mice, 6–8 weeks old, were purchased from Beijing Vital River Laboratory (Beijing, China). DDX3X^fl/fl^ mice and hepatocyte-specific DDX3X-deficient (DDX3X^ΔHep^) mice (C57BL/6J background) were generated via CRISPR/Cas9-mediated genome engineering. We identified that the DDX3X gene (NCBI reference sequence: NM_010028; ensemble: ENSMUSG00000000787) contains 17 exons, and exons 4–10 was selected as the target site. Cas9 and gRNA were co-injected into fertilized eggs for KO mouse production. The pups were genotyped by PCR followed by sequencing analysis. An overview of the targeting strategy is shown in Supplementary Fig. 2A. The sequences of the gRNAs were as follows: gRNA1: ACTTTGTATTGGGAGCATAT-AGG; gRNA2: TATTGCCCCAGCCATCATCA-TGG. Hepatic DDX3X expression was examined in DDX3X^fl/fl^ and DDX3X^ΔHep^ mice, and the results revealed a significant decrease in DDX3X^ΔHep^ mice (Supplementary Fig. 2B).

The mice were intravenously injected with Con A (C2010, Sigma‒Aldrich) at a dose of 20 mg/kg body weight at different time points. DDX3X^fl/fl^ mice and DDX3X^ΔHep^ mice received Con A (20 mg/kg) for 8 h to induce liver injury. 4-PBA (HY-A0281, MedChemExpress, 100 mg/kg) was administered intraperitoneally to the mice to inhibit ER stress. AAV-DDX3X or AAV-NC (10^12/mouse) was intravenously administered to the mice to upregulate hepatic DDX3X expression. Two weeks later, the effects of AAV-DDX3X were assessed (Supplementary Fig. 5B). Mouse models of liver ER stress of different severities were developed by the intraperitoneal injection of saline or tunicamycin (TM; Sigma, St. Louis, MO, USA) at 2.5 mg/kg for 3 h, 6 h, 12 h, or 24 h. To determine the impact of DDX3X on ER stress-induced liver injury, DDX3X-specific siRNA or NC siRNA was injected in vivo, and the mice were treated with 2.5 mg/kg TM for 24 h.

Serum samples were collected for aminotransferase (ALT) and aspartate transaminase (AST) measurements. Liver tissue samples were collected, snap-frozen in liquid nitrogen and then transferred to -80°C for further analysis. A portion of the liver was fixed in 4% paraformaldehyde for subsequent pathological analysis.

The mice were kept in the Capital Medical University animal facility under specific pathogen-free conditions and had ad libitum access to food and water. The number of animals in each group was selected according to the 3R principles of the Animal Ethics Committee and by reference to ARRIVE (Animal Research: Reporting of In Vivo Experiments) Guidelines. Mice in each group were allocated according to experimental needs using simple random sampling. All animal experiments were performed in a double-blind manner. All experiments were performed in strict accordance with the ethical guidelines of the Capital Medical University Animal Experimentation Committee and were in full compliance with the National Institutes of Health Guide for the Care and Use of Laboratory Animals (approval number: AEEI-2020-009).

### Cell culture and treatment

Primary hepatocytes were isolated from C57BL/6 mice and cultured as previously described. Specifically, the portal vein was exposed in the abdominal cavity of each mouse and perfused with 20 ml of 37 °C Hank’s balanced salt solution (HBSS, Gibco, Rockville, MD, USA) containing 0.5 mM ethylene glycol tetraacetic acid (EGTA) and 2 mg/ml bovine serum albumin (BSA, Sigma) until the liver began to flush. Then, the cells were perfused with 20 ml of HBSS containing 0.6 mg/ml collagenase type I (Sigma) and 0.01 M CaCl2. Next, the liver was excised from the abdominal cavity and transferred to a sterile 6 cm dish. The liver was rinsed with 4 ml of HBSS containing 2 mg/ml BSA, 0.01 M CaCl2 and DNase I (Millipore, Billerica, MA, USA) at 12.5 ng/ml. The liver tissue was shaken with sterile forceps, and the suspension was filtered through a 70 µm sieve (BD Biosciences, San Jose, CA, USA). Then, the cells were centrifuged at 50 × *g* for 5 min and washed 3 times with Dulbecco’s modified Eagle’s medium (DMEM, Gibco) supplemented with 10% fetal bovine serum (FBS, Gibco). Finally, approximately 30*10^6^ cells were obtained and cultured in collagen type I-coated dishes for subsequent experiments. The primary hepatocytes were incubated with DMSO or TM (20 µg/ml) for 3 h, 6 h, 12 h, or 24 h to induce different durations of ER stress.

### Statistical analysis

All the values are presented as the mean ± standard deviation (mean ± S.D.). The study materials of the two groups were controlled for factors other than the study variables before comparison to ensure that the data from the two groups were comparable. Differences between groups were analyzed via unpaired *t* tests or one-way ANOVA with GraphPad Prism 8.0 software (GraphPad Software, Inc., USA), and a *p* value < 0.05 was considered to indicate statistical significance.

Other methods are detailed in the Supplementary Materials.

## Results

### Hepatic DDX3X expression increases and DDX3X translocates into the nucleus during Con A-induced liver injury

**Fig. 1:**
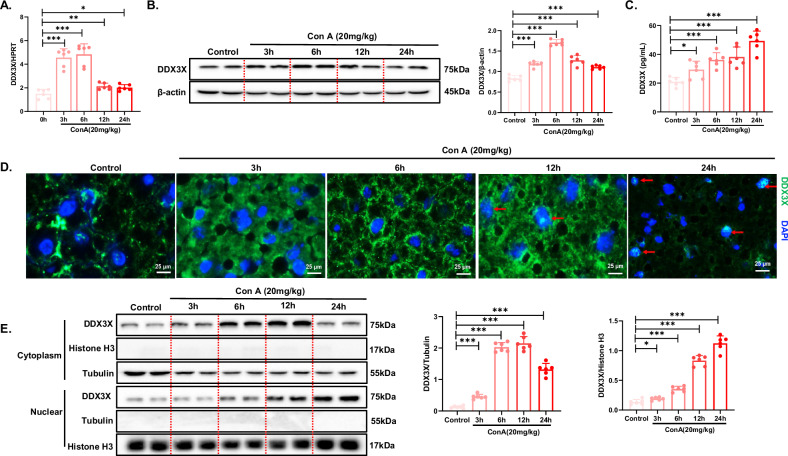
Hepatic DDX3X expression increases and DDX3X translocates into the nucleus during Con A-induced liver injury. (*P < 0.05, **P < 0.01, ***P < 0.001). C57BL/6J mice (*n* = 6/per group) were injected with Con A (20 mg/kg) for the indicated durations via the tail vein. **A** mRNA levels of DDX3X were assessed by RT‒PCR. **B** The protein levels of DDX3X were examined via WB, and densitometry was measured via ImageJ software. **C** The serum levels of DDX3X in different groups were examined via ELISA. **D** The cellular localization of DDX3X in hepatocytes was observed via immunofluorescence staining. **E** The expression level of DDX3X in the cellular fractions was examined via WB, and densitometry was measured via ImageJ software. The blots and images are representative of three independent experiments. All data from three independent experiments are shown.

Mouse models of liver injury were generated by administering Con A at different time points (Supplementary Fig. 1). The mRNA and protein levels of DDX3X significantly increased with short-term Con A stimulation (3 h, 6 h) but were reduced at later stages of liver injury (Fig. 1A, B). Concurrently, analysis of serum samples from mice revealed a progressive increase in DDX3X levels following Con A treatment (Fig. 1C). Furthermore, immunofluorescence staining revealed a significant increase in DDX3X nuclear accumulation with prolonged Con A stimulation (Fig. 1D). Moreover, we observed a gradual increase in the DDX3X level within the nucleus, peaking during the early stages of prolonged Con A treatment (Fig. 1E). Conversely, DDX3X levels in the cytoplasm exhibited a distinct pattern, peaking early during the treatment period. These data indicate that the increase in the expression level of DDX3X in liver tissues during the early stages of Con A-induced liver injury is accompanied by the translocation of this protein from the cytoplasm to the nucleus.

### Hepatocyte-specific knockout of DDX3X attenuates Con A-induced liver injury in mice

**Fig. 2:**
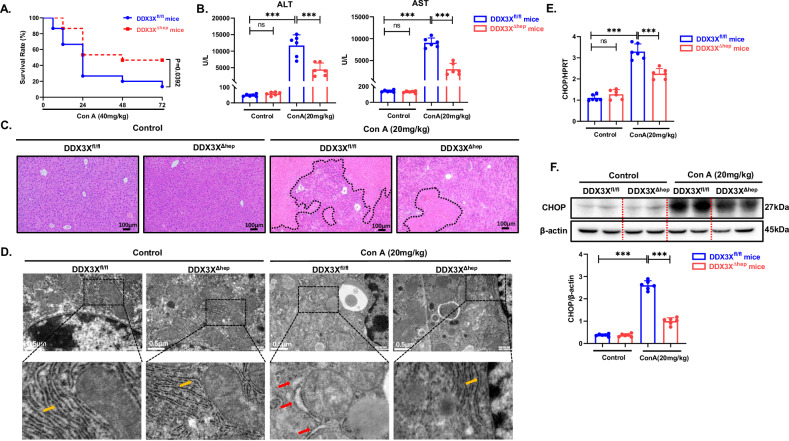
Hepatocyte-specific knockout of DDX3X attenuates Con A-induced liver injury in mice. (*P < 0.05, **P < 0.01, ***P < 0.001). **A** DDX3X^fl/fl^ and DDX3X^ΔHep^ mice were treated with Con A for the indicated durations to induce liver injury. Survival rates of DDX3X^fl/fl^ (*n* = 18) and DDX3X^ΔHep^ mice (*n* = 18) treated with 40 mg/kg Con A for 72 h. **B**–**E** DDX3X^fl/fl^ and DDX3X^ΔHep^ mice(*n* = 6/per group) were treated with Con A for 8 h to induce liver injury. **B** Liver injury was assessed by AST and ALT measurements. **C** Pathological analysis of mouse livers via H&E staining. **D** Morphological structure of endoplasmic reticulum observed under electron microscope, red arrows indicate the morphology of ER in ER stress state and yellow arrows indicate the morphology of ER in normal state. **E** mRNA levels of CHOP were assessed by RT‒PCR. **F** The protein levels of CHOP were examined via WB, and densitometry was measured via ImageJ software. The blots and images are representative of 3 independent experiments. All data from 3 independent experiments are shown.

To further elucidate the role of DDX3X in Con A-induced liver injury, we induced acute liver injury in both DDX3X^fl/fl^ mice and DDX3X^ΔHep^ mice through Con A administration (Supplementary Fig. 2C). DDX3X^ΔHep^ mice presented a significantly greater survival rate than did DDX3X^fl/fl^ mice when subjected to a lethal dose (40 mg/kg) of Con A (Fig. 2A). Notably, DDX3X^ΔHep^ mice presented substantially lower serum ALT and AST levels than did DDX3X^fl/fl^ mice following 8 h of Con A-induced liver injury, and histological analysis revealed a marked reduction in hepatocyte apoptosis and inflammatory cell infiltration in DDX3X^ΔHep^ mice (Fig. 2B, C). Taken together, our findings suggest that hepatocyte-specific knockout of DDX3X significantly mitigates Con A-induced acute liver injury in mice.

### The ER stress-DDX3X pathway promotes Con A-induced liver injury

**Fig. 3:**
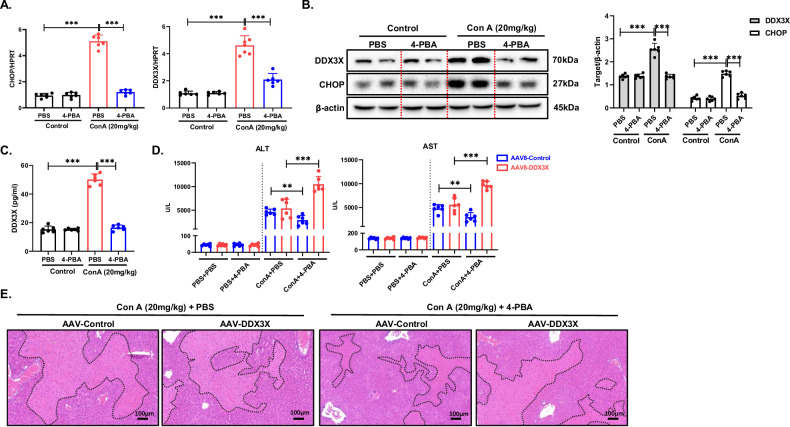
The ER stress-DDX3X pathway promotes Con A-induced liver injury. (*P < 0.05, **P < 0.01, ***P < 0.001). **A** mRNA levels of CHOP and DDX3X were assessed via RT‒PCR. **B** The protein levels of CHOP and DDX3X were examined via WB, and densitometry was measured via ImageJ software. **C** Serum levels of DDX3X in different groups were examined via ELISA. **D**, **E** C57BL/6J mice (*n* = 6/per group) were pretreated with AAV-DDX3X or AAV-NC for 2 weeks to upregulate DDX3X expression and then injected with 4-PBA (100 mg/kg) 1 h before treatment with Con A (20 mg/kg) for 8 h. **D** Liver injury was assessed by AST and ALT measurements. **E** Pathological analysis of mouse livers via H&E staining. The blots and images are representative of three independent experiments. All data from three independent experiments are shown.

Notably, we assayed other immune-related cell death pathways such as pyroptosis, necroptosis, and ferroptosis and found that the effect of DDX3X deletion on endoplasmic reticulum stress was more significant, thus further exploring CHOP-mediated apoptosis (Supplementary Fig. 3). In addition, we observed the abnormal state of swelling and enlargement of the endoplasmic reticulum was significantly diminished under electron microscopy (Fig. 2D) and a substantial reduction in the expression of CHOP in the DDX3X^ΔHep^ mice compared with the DDX3X^fl/fl^ mice treated with Con A (Fig. 2E, F). To further investigate this association, we inhibited ER stress via the use of 4-phenylbutyric acid (4-PBA) in mice with Con A-induced liver injury (Supplementary Fig. 4). Following Con A stimulation, mice pretreated with 4-PBA presented significant decreases in serum ALT and AST levels and substantial damage, accompanied by a notable decrease in inflammatory cell infiltration (Supplementary Fig. 4). These findings strongly suggest that the inhibition of ER stress provides significant protection against Con A-induced liver injury. Intriguingly, the inhibition of ER stress not only decreased the expression of DDX3X but also reduced the expression of CHOP in Con A-induced liver injury (Fig. 3A, B). Additionally, we observed a significant reduction in the serum DDX3X level in the mice pretreated with 4-PBA (Fig. 3C).

To elucidate the role of DDX3X in Con A-induced liver injury, we used adeno-associated virus (AAV) constructs to overexpress DDX3X. Mice were administered either AAV-DDX3X or AAV-control and subsequently subjected to liver injury induced by Con A (Supplementary Fig. 5A, B). These results indicate that the overexpression of DDX3X negated the protective effect of 4-PBA against Con A-induced liver injury. This is evident from the elevated levels of serum aminotransferases and the increased area of injury in mice overexpressing DDX3X (Fig. 3D, E and Supplementary Fig. 5C).

These findings suggest that DDX3X promotes Con A-induced liver injury by regulating ER stress-induced apoptosis.

### Hepatic DDX3X expression increases and DDX3X translocates into the nucleus during ER stress-induced liver injury

**Fig. 4:**
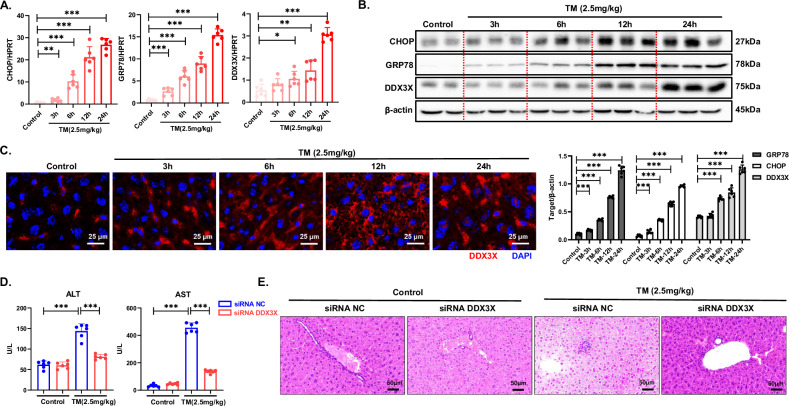
Hepatic DDX3X expression increases and DDX3X translocates into the nucleus during ER stress-induced liver injury. (*P < 0.05, **P < 0.01, ***P < 0.001). **A**–**C** C57BL/6J mice (*n* = 6/per group) were intraperitoneally injected with TM for 3 h, 6 h, 12 h, or 24 h to induce different degrees of ER stress in vivo. **A** mRNA levels of GRP78, CHOP, and DDX3X were assessed via RT‒PCR. **B** The protein levels of GRP78, CHOP, and DDX3X were examined via WB. **C** The cellular localization of DDX3X in hepatocytes was observed via immunofluorescence staining. **D**, **E** C57BL/6J mice (*n* = 6/per group) were treated with siRNA NC or siRNA DDX3X in vivo and injected with TM for 24 h. **D** Liver injury was assessed by AST and ALT measurements. **E** Pathological analysis of mouse livers via H&E staining. The blots and images are representative of three independent experiments. All the data from three independent experiments are shown.

To further investigate the precise mechanism by which DDX3X mediates ER stress-induced apoptosis, we induced liver injury in mice through the intraperitoneal injection of tunicamycin (TM) at various time points (Supplementary Fig. 6A). Gradual increases in serum ALT and AST levels were observed, concomitant with liver injury, as evidenced by H&E staining following prolonged TM treatment (Supplementary Fig. 6B, C). The results revealed a significant increase in the expression of CHOP and GRP78 with TM treatment. Notably, both the mRNA and protein levels of DDX3X gradually increased under prolonged ER stress conditions (Fig. 4A, B). Additionally, DDX3X translocated into the nucleus with prolonged ER stress (Fig. 4C). Collectively, these findings demonstrate a significant increase in the expression level of DDX3X and its translocation into the nucleus during ER stress-induced liver injury in mice. C57BL/6 mice were subsequently treated with either DDX3X siRNA or NC siRNA (Supplementary Fig. 7) followed by the intraperitoneal injection of TM for 24 h. Serum ALT and AST levels and H&E staining revealed that DDX3X siRNA administration protected against ER stress-induced liver injury (Fig. 4D, E). These findings collectively underscore the critical role of DDX3X as a regulator in promoting cell apoptosis induced by ER stress.

### ER stress increases DDX3X expression and promotes the cytoplasmic–nuclear transport of DDX3X in vitro

**Fig. 5:**
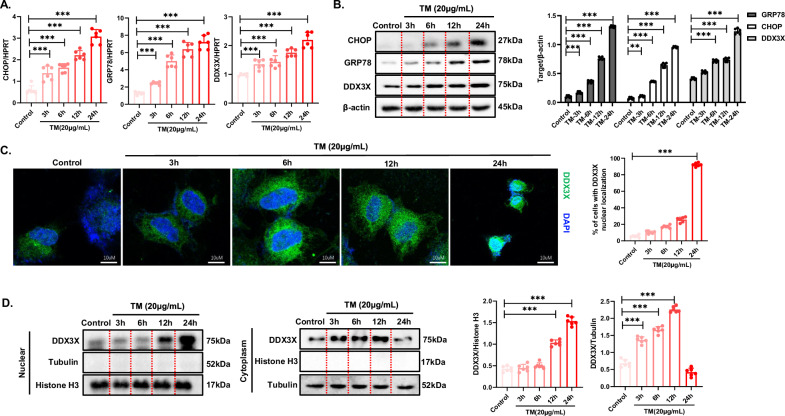
ER stress increases DDX3X expression and promotes the cytoplasmic–nuclear transport of DDX3X in vitro. (*P < 0.05, **P < 0.01, ***P < 0.001). Primary hepatocytes were treated with TM for 3 h, 6 h, 12 h, or 24 h to induce different durations of ER stress. **A** mRNA levels of GRP78, CHOP, and DDX3X were assessed via RT‒PCR. **B** GRP78, CHOP, and DDX3X in whole-cell lysates were examined via WB, and densitometry was performed with ImageJ software. **C** The expression of DDX3X in primary hepatocytes was observed via confocal microscopy. **D** The expression level of DDX3X in the cellular fractions was examined via WB. The blots and images are representative of three independent experiments. All data from three independent experiments are shown.

To explore the detailed mechanisms by which DDX3X regulates ER stress-induced apoptosis in detail, primary hepatocytes were subjected to ER stress induced by TM for various durations. The expression levels of GRP78 and CHOP gradually increased, indicating increased ER stress severity (Fig. 5A, B). Furthermore, both the mRNA and protein levels of DDX3X tended to increase with prolonged ER stress (Fig. 5A, B). Notably, confocal analysis revealed increased nuclear translocation of DDX3X following prolonged ER stress (Fig. 5C). Consistently, the results demonstrated peak nuclear localization of DDX3X in primary hepatocytes stimulated with TM for 24 h. Conversely, the cytoplasmic fractions presented a gradual increase in DDX3X levels during the early stages (TM treatment for 3 h, 6 h, and 12 h), followed by a decrease in the late stage (TM treatment for 24 h) of ER stress (Fig. 5D). Therefore, the expression level of DDX3X gradually increases and DDX3X translocates into the nucleus in response to prolonged ER stress.

### The nuclear translocation of DDX3X promotes cell apoptosis under ER stress

To investigate the functional impact of DDX3X during ER stress, we used specific siRNAs to knock down DDX3X expression in primary hepatocytes, followed by treatment with TM for 24 h. Cell viability analysis revealed a significant increase in cell survival rates following TM treatment in cells with reduced DDX3X expression (Supplementary Fig. 8A). Furthermore, LDH assays demonstrated a notable decrease in cell death rates under ER stress conditions upon DDX3X knockdown (Supplementary Fig. 8A). Consistent with these findings, flow cytometry analysis of cell apoptosis rates revealed a reduction in the degree of apoptosis in response to ER stress following DDX3X knockdown (Supplementary Fig. 8B). These findings collectively suggest a role for DDX3X in promoting cell apoptosis under ER stress conditions.

**Fig. 6:**
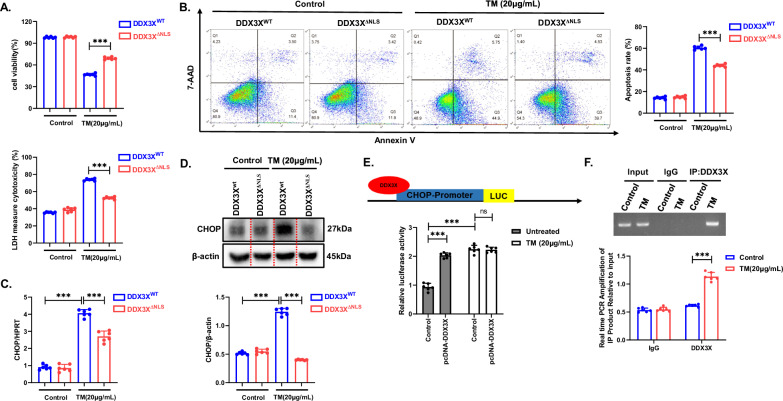
The nuclear translocation of DDX3X transcriptionally activates CHOP to promote cell apoptosis under ER stress. (*P < 0.05, **P < 0.01, ***P < 0.001). Primary hepatocytes were transfected with DDX3X^ΔNLS^ or DDX3X^wt^ plasmids before TM stimulation for 24 h. **A** Cell viability was measured by a CCK-8 assay. Cell death was examined by LDH release. **B** Cell apoptosis was measured by flow cytometry. The cells were recognized as different populations according to their staining: the lower left quadrant was identified as live cells, the lower right quadrant was identified as early apoptotic cells, the upper left quadrant was identified as necrotic cells, and the upper right quadrant was identified as late apoptotic cells. The percentage of apoptotic cells was calculated as the sum of early and late apoptotic cells. **C** mRNA levels of CHOP were assessed by RT‒PCR. **D** The expression level of CHOP was examined via WB. **E** Primary hepatocytes were first transfected with negative or DDX3X-overexpressing plasmids. CHOP promoter-luciferase reporter plasmids were transfected into the indicated primary hepatocytes, which were then treated with TM for 24 h. Luciferase activity was measured. **F** ChIP analysis of DDX3X binding to the CHOP promoter in primary hepatocytes treated with TM or DMSO for 24 h. Bar graphs show the amount of immuno-precipitated DNA as detected by RT-PCR analysis. The blots and images are representative of three independent experiments. All data from three independent experiments are shown.

Given the translocation of DDX3X from the cytoplasm to the nucleus in hepatocytes during prolonged ER stress, we investigated the impact of DDX3X subcellular localization on ER stress-mediated cell fate. To prevent the nuclear translocation of DDX3X, primary hepatocytes were transfected with DDX3X^ΔNLS^ plasmids during prolonged ER stress induction (Supplementary Fig. 9). The results demonstrated a significant increase in cell survival rates upon the inhibition of DDX3X nuclear translocation during ER stress (Fig. 6A). Furthermore, the reduction in cell apoptosis rates was consistent with the inhibition of DDX3X translocation to the nucleus under ER stress conditions (Fig. 6B). Furthermore, the results shows that a decrease in serum ALT/AST levels, as well as hepatocyte edema and inflammatory cell infiltration, in mice transfected with DDX3X^ΔNLS^ plasmid(expressed DDX3X that deletion of nuclear localization signal), suggested that blockade of DDX3X nuclear translocation significant alleviated TM-induced liver injury in mice (Supplementary Fig. 10). These findings suggest a crucial role for the nuclear translocation of DDX3X in ER stress-induced hepatocyte apoptosis.

### DDX3X transcriptionally activates CHOP to promote cell apoptosis under ER stress

CHOP functions as a pivotal transcription factor that participates in ER stress-induced apoptosis, and its expression is intricately regulated by diverse signaling pathways. Primary hepatocytes were subjected to siRNA transfection to downregulate DDX3X expression, and the subsequent examination of CHOP expression levels in ER stress-induced cells revealed a noteworthy decrease in both CHOP mRNA and protein levels (Supplementary Fig. 8C, D). These findings suggest that DDX3X may increase the expression of CHOP under ER stress conditions. Importantly, the inhibition of DDX3X nuclear translocation by DDX3X^ΔNLS^ during ER stress resulted in the suppression of CHOP expression (Fig. 6C, D). These results underscore the critical role of DDX3X and its nuclear translocation in modulating CHOP expression during ER stress. To further validate the regulation of CHOP by DDX3X, we conducted a luciferase reporter assay driven by the CHOP promoter to assess the transcriptional activity of CHOP. ER stress induction notably augmented luciferase activity following 24 h of TM treatment, whereas DDX3X overexpression led to increased luciferase activity even in the absence of TM stimulation (Fig. 6E). We developed ChIP assay with anti-DDX3X or IgG and quantitative PCR (qPCR) demonstrated that endogenous DDX3X occupy the promoter regions of CHOP, which was significantly induced by TM treatment in primary hepatocyte (Fig. 6F). Additionally, given the helicase activity of DDX3X, whether it regulates CHOP through this activity was verified. The helicase activity of DDX3X were inhibited by RK-33, a small-molecule inhibitor. The expression level of CHOP did not affect by RK-33 treatment, which suggests that the regulation of CHOP by DDX3X is not dependent on its helicase activity (Supplementary Fig. 11). Taken together, these results show that the translocation of DDX3X into the nucleus directly promotes the expression of CHOP at the transcription level during ER stress.

### DDX3X is increased in the liver tissue of patients with immune-mediated liver injury and serves as a potential predictive marker for LF prognosis

**Fig. 7:**
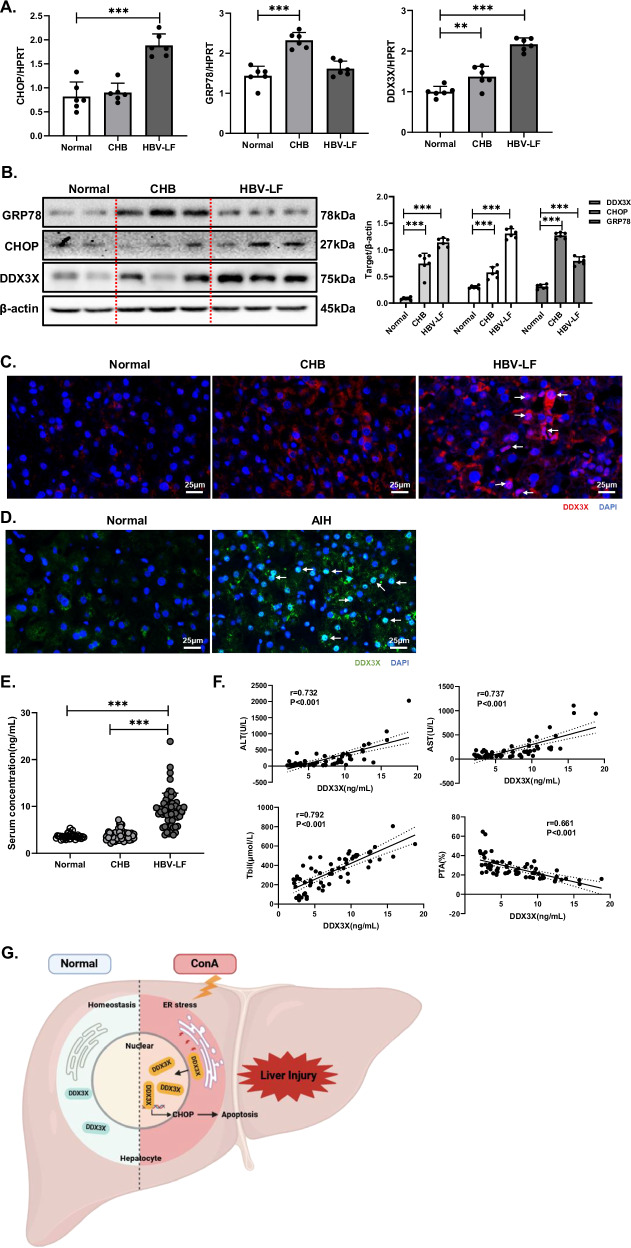
DDX3X is increased in the liver tissue of patients with immune-mediated liver injury and serves as a potential predictive marker for LF prognosis. (*P < 0.05, **P < 0.01, ***P < 0.001). Liver tissues from normal controls, patients with CHB and patients with HBV-LF were collected to examine the expression of DDX3X. **A** mRNA levels of GRP78, CHOP and DDX3X were assessed via RT‒PCR. **B** The expression levels of GRP78, CHOP and DDX3X were examined via WB. **C** The cellular localization of DDX3X in the liver tissue of different groups was determined by immunochemistry. **D** The cellular localization of DDX3X in the liver tissue of patients with AIH or normal controls was determined by immunofluorescence staining. **E** ELISA was used to examine serum DDX3X levels among 30 normal controls, 60 patients with CHB, and 60 patients with HBV-LF. **F** The correlation between DDX3X levels and liver function indexes in 60 patients with hepatitis B liver failure. **G** Schematic showing the translocation of DDX3X from the cytoplasm to the nucleus, where it activates the transcription of CHOP under ER stress conditions during Con A-induced liver injury. The blots and images are representative of 3 independent experiments. All data from 3 independent experiments are shown.

To further investigate the clinical relevance of DDX3X, we analyzed DDX3X expression patterns in healthy individuals, patients with chronic hepatitis B (CHB), and patients with hepatitis B virus-associated liver failure (HBV-LF). We assessed serum DDX3X levels in 30 healthy controls, 60 CHB patients, and 60 HBV-LF patients; detailed clinical characteristics are provided in Supplementary Table 1. The expression of CHOP and GRP78 was increased in LF patients, and the DDX3X mRNA and protein levels were significantly elevated in the liver tissues of LF patients (Fig. 7A, B). Compared with that in healthy controls and CHB patients, the nuclear translocation of DDX3X in HBV-LF patients was substantially greater (Fig. 7C). Similarly, a considerable amount of nuclear DDX3X was observed in AIH patients (Fig. 7D). These results collectively suggest that DDX3X levels are markedly increased and that DDX3X is translocated to the nucleus in patients experiencing immune-mediated liver injury.

We subsequently investigated serum DDX3X levels across various groups. The results revealed a significant increase in the serum DDX3X level among patients with HBV-related LF (Fig. 7E). Concurrently, we analyzed the correlation between DDX3X levels and liver function indexes in 60 patients with hepatitis B liver failure, which were positively correlated with ALT, AST and TBil, and negatively correlated with PTA (Fig. 7F). Furthermore, we conducted an analysis to assess the correlation between dynamic serum DDX3X levels and different prognoses among 12 LF patients. Among 8 survivors, the DDX3X level consistently decreased, whereas it rebounded in 4 patients with poor prognoses (Supplementary Fig. 12). These findings suggest that the serum DDX3X level could serve as a potential predictive factor for LF prognosis.

## Discussion

Although emerging evidence suggests that DDX3X participates in the cellular stress response [12, 13], its potential role in immune-mediated liver injury remains unclear. This study is the first to demonstrate that the hepatocyte-specific knockout of DDX3X can protect against immune-mediated liver injury. We elucidated the mechanism underlying the pivotal role of DDX3X in driving the ER stress-CHOP apoptosis pathway through its translocation from the cytoplasm to the nucleus (Fig. 7G). Our findings reveal a novel role of DDX3X in hepatocyte homeostasis: it exacerbates ER stress and promotes Con A-induced liver injury.

Several studies have provided evidence that DDX3X is involved in various liver diseases [13]. ER stress-induced hepatocyte apoptosis has been confirmed in a wide variety of immune-mediated liver injuries, including viral hepatitis B and autoimmune liver disease [14, 15]. In a previous study, we demonstrated a significant difference in the expression of ER stress markers in liver tissue during the progression from hepatitis B to ACLF [16]. Protracted ER stress initiates the apoptosis pathway via UPR signaling, and the transcription factor CHOP/GADD153 is the most well-characterized proapoptotic pathway in response to ER stress [17]. This study confirmed that DDX3X translocates into the nucleus to promote the expression of CHOP directly at the transcription level during ER stress. A previous study suggested that DDX3 is required for ER stress-induced ATF4 expression through binding with the eIF4F complex [18]. Studies have shown that CHOP transcription is primarily activated by ATF4, although other signaling pathways may contribute to its transcription [19]. However, this study revealed that DDX3X transcriptionally activates CHOP to initiate hepatocyte apoptosis under ER stress.

This study revealed that the increased nuclear translocation of DDX3X is the pivotal mechanism driving ER stress-induced apoptosis. Previous investigations have recognized DDX3X as a nucleocytoplasmic shuttling protein involved in both the nuclear and cytoplasmic stages of gene expression [20, 21]. Considering the distinct cytoplasmic and nuclear functions of DDX3X, its subcellular localization could influence downstream signaling pathways and cellular outcomes. Studies have documented the involvement of CRM-1 and TAP in regulating the export of DDX3X from the nucleus [22, 23], with the nuclear export signal (NES) being essential for the export of human DDX3X from the nucleus [24]. Intriguingly, our findings indicate that in Con A-induced liver injury, DDX3X translocates from the cytoplasm to the nucleus in hepatocytes. Furthermore, we observed that DDX3X accumulated in the nucleus in response to prolonged ER stress and revealed that the deletion of nuclear localization signal (NLS) sites significantly mitigated ER stress-induced cell apoptosis. Therefore, increased DDX3X nuclear translocation is the key mechanism driving ER stress-induced apoptosis during Con A-induced liver injury.

An unexpected finding in our study was the detection of high serum DDX3X levels in immune-mediated liver injury, which was observed both in Con A-induced mice and patients with HBV-related liver failure (LF). These findings suggest a potential role for serum DDX3X as a biomarker for diagnosing and prognosticating HBV-related LF. It has been suggested in the literature [25] that there are gender differences in DDX3X expression, and our analyses showed that serological levels of DDX3X in clinical samples from different cohorts were not statistically significant, although gender differences could be seen (Supplementary Fig. 13). Furthermore, our results revealed a gradual increase in hepatic DDX3X expression from chronic hepatitis B (CHB) to HBV-LF, an effect that was consistent with elevated levels of ER stress markers. Furthermore, we confirmed a significant increase in the serum DDX3X level in LF patients, indicating its diagnostic utility for LF. Additionally, we observed a strong association between decreased DDX3X levels and survival, with increased levels indicating a poor prognosis for patients with LF. These variations in DDX3X levels reflect disease progression, regression, and fluctuations. Our findings underscore DDX3X as an indicator of the clinical course of the disease. Of course, the sample size of this study does not confirm that DDX3X can be used as a diagnostic marker for immune liver injury, nor does it address whether future clinical applications should consider gender differences. Therefore, we will explore this issue further in subsequent research.

In conclusion, our study revealed that the cytoplasm-to-nucleus translocation of DDX3X exacerbates liver injury by activating the ER stress-CHOP pathway during immune-mediated liver injury. These novel findings suggest a paradigm in which DDX3X is a potential regulator of liver homeostasis, indicating that DDX3X is a promising target for the clinical treatment of immune-mediated liver injury.

## Supplementary information

Supplemental Materials

Original western blots

## Data Availability

The datasets generated during and/or analyzed during the current study are available from the corresponding author on reasonable request.
