# Supplementary material for: The cytoplasmic-nuclear transport of DDX3X promotes immune-mediated liver injury in mice regulated by endoplasmic reticulum stress
Source: Cell Death Dis. 2024 Sep 30;15(9):702. doi: 10.1038/s41419-024-07076-9 (PMC11442484; doi:10.1038/s41419-024-07076-9)
Supplement: Supplementary file 1 — Supplemental Materials [file 41419_2024_7076_MOESM1_ESM.pdf]

The Cytoplasmic-nuclear Transport of DDX3X Promotes Immune-mediated Liver Injury in Mice Regulated by Endoplasmic Reticulum Stress

Zihao Fan[†1], Ling Xu[†1], Yao Gao[†1], Yaling Cao[1], Yuan Tian[1], Zhenzhen Pan[1], Linlin Wei[1], Sisi Chen[1], Xiangying Zhang[1], Mei Liu[*2], Feng Ren[*1]

[1]Beijing Institute of Hepatology/Beijing Youan Hospital, Capital Medical University, Beijing 100069, China

[2]Department of Oncology, Beijing Youan Hospital, Capital Medical University, Beijing 100069, China.

Running title: ERS-DDX3X pathway Promotes Liver Injury

[†]These authors contributed equally to this work

*** Correspondence to:**

Feng Ren, Beijing Institute of Hepatology/Beijing Youan Hospital, Capital Medical University, No. 8, XitouTiao Road, Youwai Street, Fengtai District, Beijing 100069, China. Email: renfeng7512@ccmu.edu.cn

Mei Liu, Department of Oncology, Beijing Youan Hospital, Capital Medical University, No. 8, Xitou Tiao Road, Youwai Street, Fengtai District, Beijing 100069, China. Email: liumei@ccmu.edu.cn

Supplemental materials and methods

Histological and biochemical assessment

Liver tissue was collected as described above, sectioned after being embedded in paraffin and then stained with hematoxylin and eosin (H&E). Images of histological injury to liver tissue were obtained and observed with a Leica DM2500 fluorescence microscope system.

Serum ALT and AST levels were measured with a Hitachi 7600 automatic biochemical analysis system. Serum samples were diluted three times before analysis and analyzed following the manufacturer's standard protocols.

Real-time reverse transcription–polymerase chain reaction (RT–PCR)

Total RNA was extracted from cells or liver tissue using TRIzol reagent. cDNA was subsequently obtained via reverse transcription using the PrimeScript™ RT reagent Kit (TaKaRa, Kusatsu, Shiga, Japan) according to the manufacturer's instructions. Specifically, the reaction mixture included 2 µl of 5X PrimeScript Buffer, 0.5 µl of PrimeScript RT Enzyme Mix I, 0.5 µl of oligo dT primer (50 µM), 0.5 µl of random 6-mers (100 µM), 500 ng of total RNA, and 10 µl of RNase-free dH2O. The reverse transcription reaction conditions were as follows: 37°C for 15 min, 85°C for 5 sec, and hold at 4°C.

Real-time PCR was conducted with 10 µl of SYBR mixture, 0.4 µl of forward primer (10 µM), 0.4 µl of reverse primer (10 µM), 4 µl of cDNA, and RNase-free dH2O up to 20 µl. The reaction conditions were as follows: 50°C for 2 min and 95°C for 5 min, followed by 50 cycles of 95°C for 15 s and 60°C for 30 s. All the primers for specific targets are presented in Supplemental Table 3. The relative level of target mRNA expression was normalized to that of HPRT and was calculated using the $2^{-\Delta\Delta Ct}$ method.

Western blotting

RIPA buffer containing 1% protease and phosphatase inhibitors was used to extract total protein from primary hepatocytes and liver tissue. A Pierce™ Rapid Gold BCA Protein Assay Kit (Invitrogen, Thermo Fisher Scientific) was used to measure the protein concentrations according to the manufacturer's instructions. Then, a 20 μg–40 μg protein sample was separated via SDS–12% polyacrylamide gel electrophoresis and transferred onto polyvinylidene fluoride (PVDF) membranes (Bio–Rad, Hercules, CA). The membranes were subsequently blocked with 5% skim milk at room temperature for 1 h and incubated with antibodies against different targets at 4°C overnight. All the primary antibodies used in this study are detailed in Supplemental Table 4. Then, the membranes were incubated with horseradish peroxidase-conjugated anti-mouse IgG (1:2000, Cell Signaling Technology, Cat# 7076) or anti-rabbit IgG (1:1000, Cell Signaling Technology, Cat# 7074) at temperature for 1 h.

Finally, Super Signal™ West Pico PLUS (Invitrogen, Thermo Fisher Scientific) was used to develop the blots, which were visualized with a chemiluminescence detection system (Bio-Rad). The grayscale values of the bands of target proteins were measured using ImageJ software (National Institutes of Health, Bethesda, USA).

Immunofluorescence

Primary hepatocytes were cultured and treated with TM as previously described. The cells were fixed in 4% paraformaldehyde at room temperature for 20 min and then treated with 0.5% Triton X-100 at 37°C for 20 min. Next, the cells were blocked in 3% BSA containing 1% goat serum at 37°C for 60 min. Then, the cells were incubated at 4°C overnight

with anti-DDX3X (1:500, Abcam, Cambridge, UK) or anti-PPARα (1:500, Abcam) antibodies diluted in 3% BSA, followed by incubation at 37°C for 30 min with Alexa Fluor 488 goat anti-rabbit IgG (1:200, Invitrogen, Thermo Fisher Scientific) or Alexa Fluor 568 goat anti-mouse IgG (1:200, Invitrogen, Thermo Fisher Scientific) diluted in 3% BSA. Finally, 10 µl of DAPI (Abcam) was used to stain the nuclei. Images were acquired using a Leica confocal microscope system (Leica TCS SP8).

Cell counting kit-8 (CCK-8) assay

Primary hepatocytes were seeded in a 96-well plate and treated as described previously. A CCK-8 assay kit (Sigma, Cat# 96992) was used to assess cell viability. After drug exposure for the appropriate time, CCK-8 solution was added directly to the medium, and the cells were incubated in a cell culture chamber for 1 h. Then, the absorbance of each well was assessed at 490 nm. The ratio of the absorbance of the experimental group to that of the untreated group was calculated as the percentage of viable cells.

Cytotoxicity lactate dehydrogenase (LDH) assay

Primary hepatocytes were seeded in a 96-well plate and treated as described previously. A cytotoxicity LDH assay kit (Sigma, Cat# 91963) was used to determine cytotoxicity by measuring LDH activity released from damaged cells. After drug treatment for the appropriate time, the maximum amount of enzyme release reagent was added 1 h before incubation with the working solution. After incubation with the reagent for approximately 1 h in the dark, the absorbance of each well was measured at 490 nm. The ratio of the absorbance of the experimental group to that of the maximum enzyme release group was calculated as the percentage of cell death.

Flow cytometry

Primary hepatocytes were plated in 6-well plates at a density of 1×10^6 cells per well and treated as described previously. An Annexin V-phycoerythrin (PE)/7-amino-actinomycin (7-AAD) double-staining kit (BD Bioscience, Cat# 559763) was used to assess cell apoptosis. After exposure to drugs, the cells were washed with PBS buffer and resuspended in 100 µl of binding buffer containing 10 µl of PE and 7-AAD. The samples were subsequently analyzed with a Calibur flow cytometer (BD Bioscience), and the data were analyzed with FlowJo software. The cell apoptosis rate was determined as the sum of late-phase apoptotic cells (PE positive and 7-AAD positive) and early-phase apoptotic cells (PE positive and 7-AAD negative).

siRNA and plasmid transfection

To investigate the role of DDX3X during ER stress, primary hepatocytes were transfected with siRNA oligonucleotides against DDX3X (5 µM) or the negative control (5 µM) 24 h before TM stimulation. All siRNA oligonucleotides were purchased from GenePharma (Shanghai, China); detailed information is presented in **Supplemental Table 1.** siRNA transfection was performed according to the protocol provided with Lipofectamine 2000 (Invitrogen, Thermo Fisher Scientific, Carlsbad, CA). Specifically, for the transfection mixture for one well of a 6-well plate with 70% confluent cells, 9 µl of Lipofectamine® and 2 µg of siRNA were diluted in 150 µl of Opti-MEM®. The two dilutions were subsequently combined and incubated for 5 min at room temperature. Finally, a total volume of 250 µl of the transfection mixture was added dropwise to each well, and the cells were then incubated for 24 h. The efficiency of the siRNA for specific targets was determined via Western blot assays.

To prevent the nuclear translocation of DDX3X, we generated plasmids in which the nuclear localization signal (NLS) within the N-terminus and N-terminal tail (1-264), which is responsible for the localization of DDX3X, was deleted. Plasmid transfection was performed according to the protocol provided with Lipofectamine 2000 (Invitrogen, Thermo Fisher Scientific).

Nuclear/cytosolic fractionation

Liver tissue was collected following previously established procedures, or primary hepatocytes were subsequently prepared in 6 cm dishes and treated as indicated. A nuclear/cytosol fractionation kit (Abcam, Cat# ab289882) was used to extract cytoplasmic and nuclear proteins. The cells were collected and incubated in cytosol extraction buffer A (CEB-A) for 10 min on ice and then in cytosol extraction buffer B (CEB-B) for 1 min. Then, the lysate was centrifuged, and the supernatant was transferred to a new centrifuge tube containing the cytoplasmic extract for further use. The pellet was subsequently resuspended in 100 µl of ice-cold nuclear extraction buffer (NEB), incubated on ice for 40 min, and centrifuged, after which the supernatant was stored as the nuclear extract. The cytoplasmic and nuclear proteins were stored at -80°C for further Western blot analysis.

Dual-luciferase reporter assay

Primary hepatocytes were cultured in a 24-well plate and transfected with the corresponding plasmids described below. The transfection procedure was the same as that described previously. Specifically, a CHOP-promoting plasmid (pGL4) or negative control plasmid (pGL4) was transfected into cells. Moreover, the pRL-TK vector containing Renilla luciferase was cotransfected into cells as a positive control. After the plasmids were

transfected for 36 h, primary hepatocytes were treated with DMSO or TM (20 µg/ml) for 24 h.

After the cells were treated with drugs, they were assessed with luminometers and a Dual-Luciferase® Reporter Assay Kit (Promega Corporation, Wisconsin, USA). For the cells grown in the 24-well plate, 100 µl of passive lysis buffer was added to each well and incubated at room temperature for 15 min. Then, 20 µl of cell lysate was transferred to a 96-well plate and mixed with 100 µl of Luciferase Assay Reagent II. With a plate-reading luminometer, we set a 2-s delay and a 10-s readout for firefly luciferase activity. Then, 100 µl of Stop & Glo® Reagent was added to each well, and the Renilla luciferase activity was measured with a 2-s delay and a 10-s read time.

ChIP assay

Primary hepatocytes were treated with TM for 24 h, followed by crosslinking with 1% formaldehyde for 15 min at room temperature and subsequent ChIP using anti-DDX3X, the detailed procedure was performed as the instructions of Pierce Agarose ChIP Kit (Thermo Scientific™ 26156). Purified immunoprecipitated DNA was analysed by qPCR, primers used for qPCR are listed in Supplementary Table 3.

Supplemental Figures

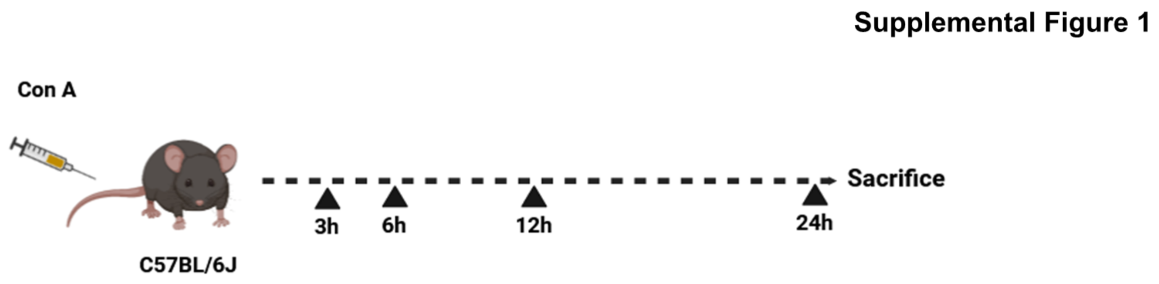

Supplemental Figure 1

Schematic diagram of Con A-induced liver injury in mice. C57BL/6J mice were treated with Con A (20 mg/kg) via tail vein injection for the indicated durations.

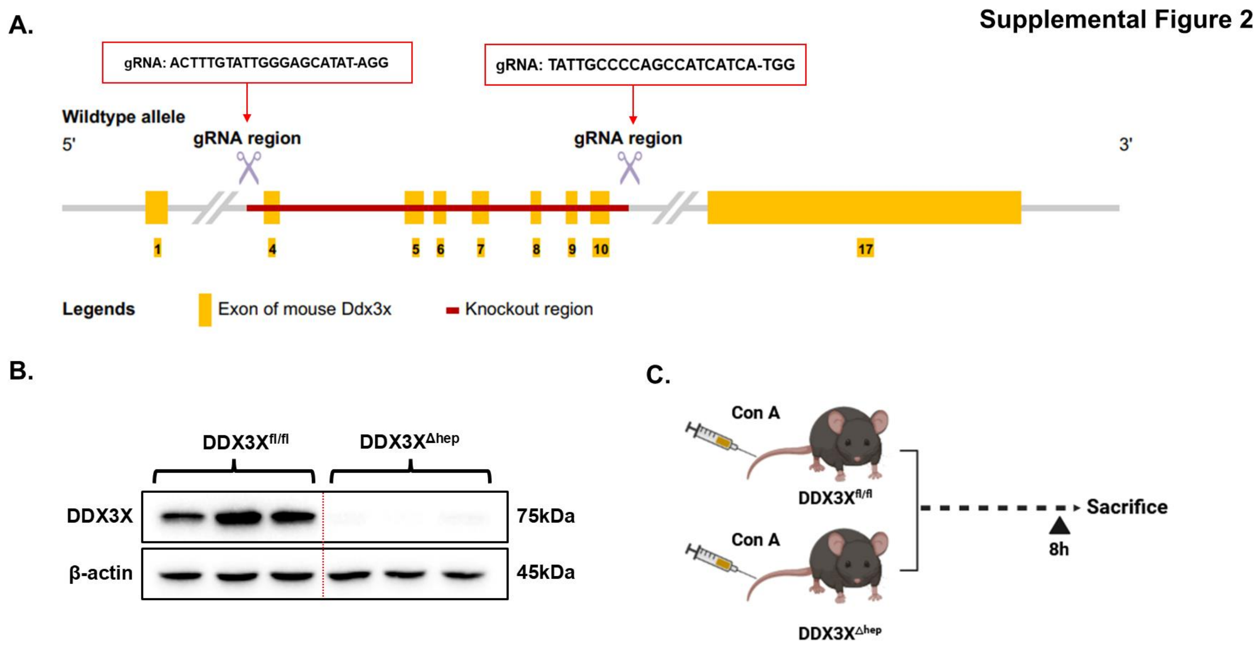

Supplemental Figure 2

A. Overview of the targeting strategy for generating DDX3X$^{\Delta Hep}$ mice through CRISPR-Cas9. **B.** The expression of DDX3X was examined in DDX3X$^{fl/fl}$ and DDX3X$^{\Delta Hep}$ mice. **C.** DDX3X$^{fl/fl}$ and DDX3X$^{\Delta Hep}$ mice were treated with Con A (20 mg/kg) via tail vein injection

for 8 h.

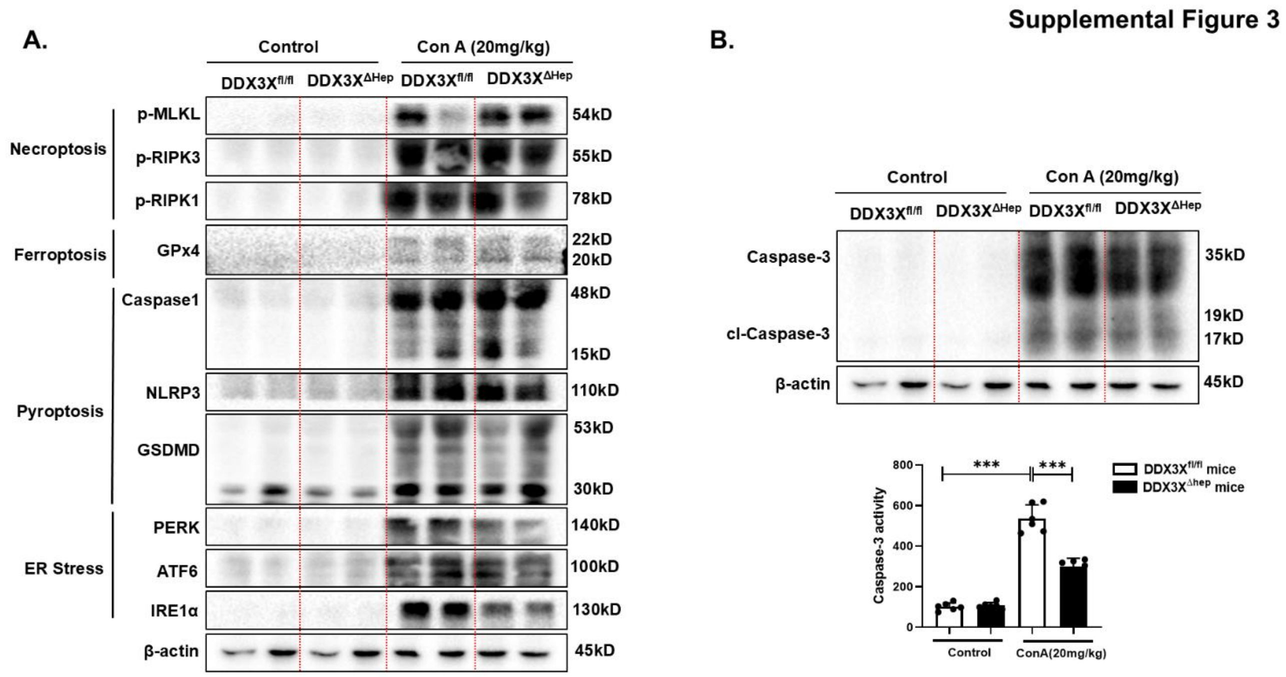

Supplemental Figure 3

A. The assays of the key molecules about immune-related cell death pathways such as pyroptosis, necroptosis and ferroptosis. **B.** The caspase activity and expression changes in ConA-induced liver injury of mice. *** denotes a p value of <0.001.

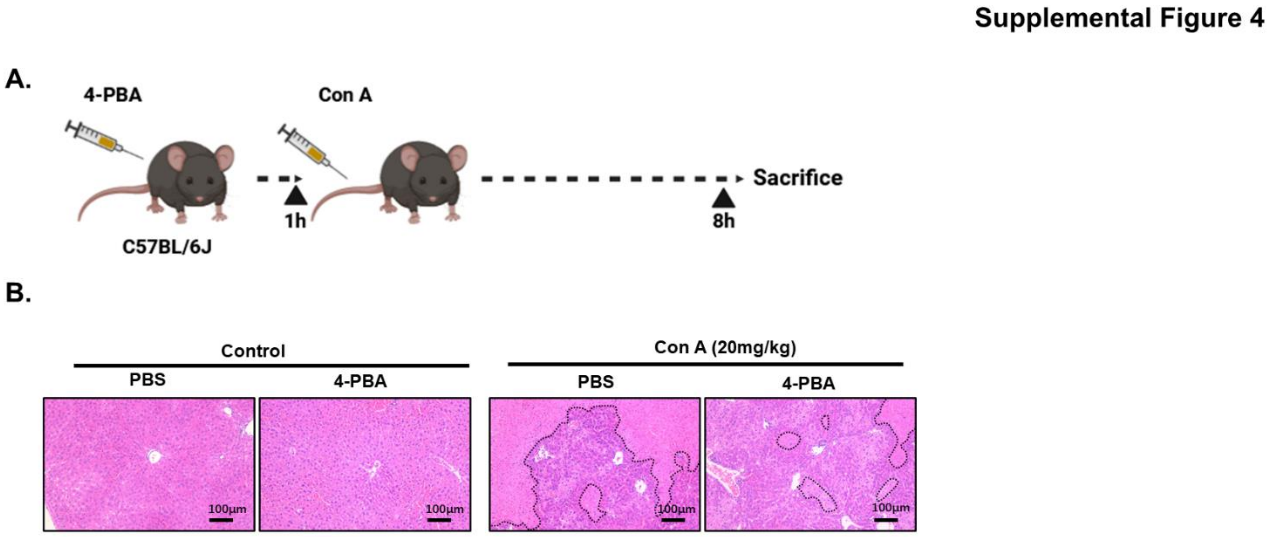

Supplemental Figure 4

A. Schematic diagram of Con A-induced liver injury in mice. C57BL/6J mice were intraperitoneally injected with 4-PBA (100mg/kg) before Con A (20 mg/kg) administration. **B.** Pathological analysis of mouse livers via H&E staining.

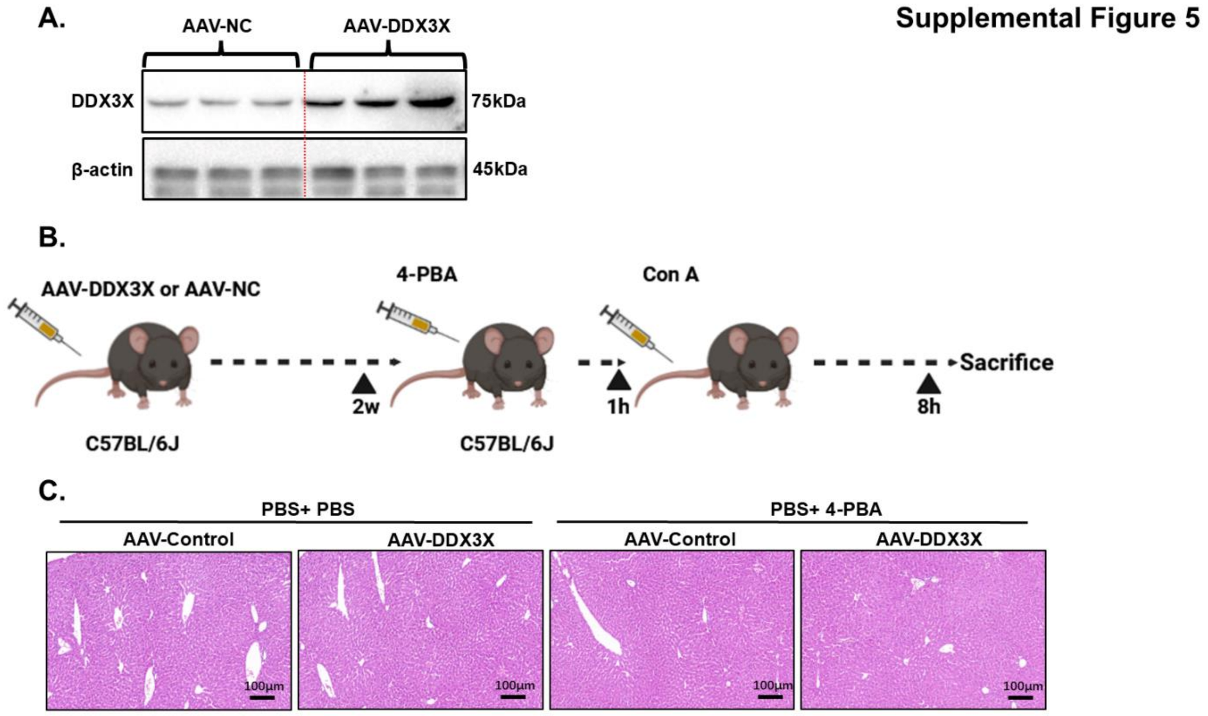

Supplemental Figure 5

A. AAV-DDX3X or AAV-NC (10^12/mouse) was intravenously administered to the mice to upregulate hepatic DDX3X expression. Two weeks later, the efficiency of AAV-DDX3X were assessed from the expression of DDX3X in liver tissues. **B.** C57BL/6J mice were treated with AAV-DDX3X or AAV-NC for 2 weeks and then intraperitoneally injected with 4-PBA before Con A (20 mg/kg) administration. **C.** Pathological analysis of mouse livers via H&E staining.

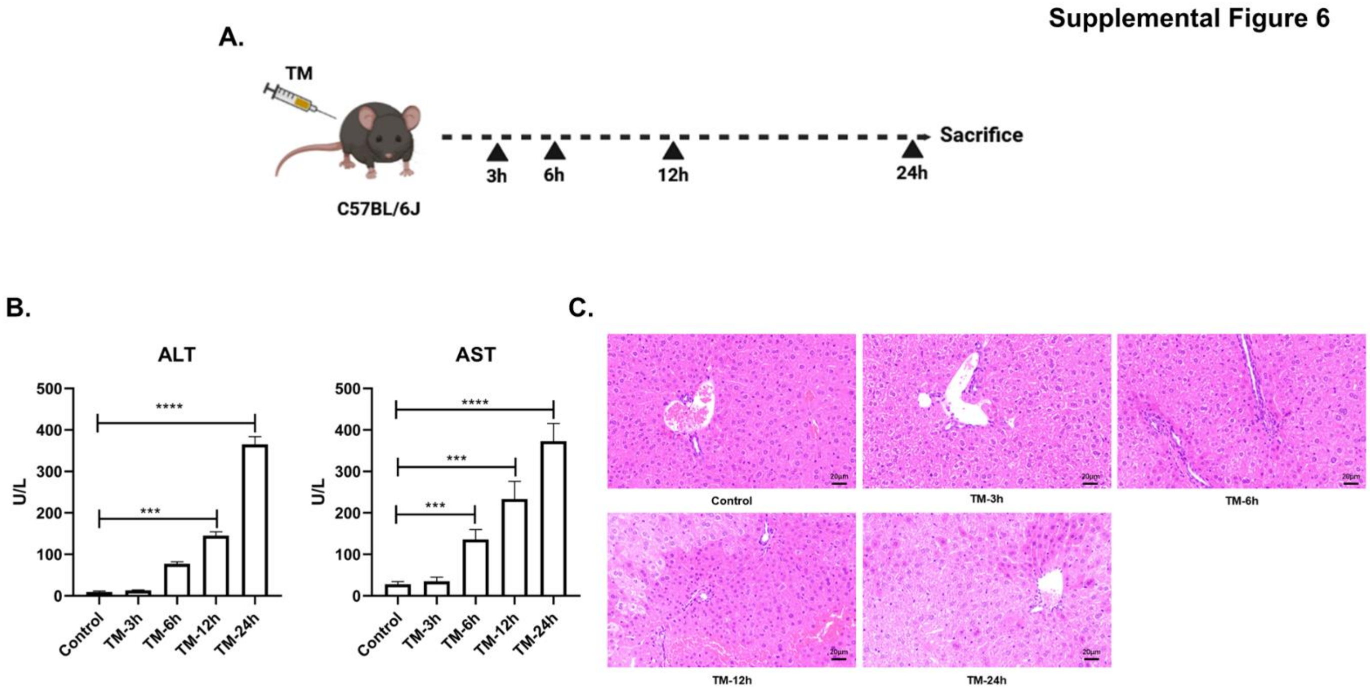

Supplemental Figure 6

A. Schematic diagram of TM-induced liver injury in mice. C57BL/6J mice were intraperitoneally injected with TM (2.5 mg/kg) for the indicated durations. **B.** Liver injury was assessed by AST and ALT measurements. **C.** Pathological analysis of mouse livers via H&E staining. **** denotes a p value of <0.0001.

Supplemental Figure 7

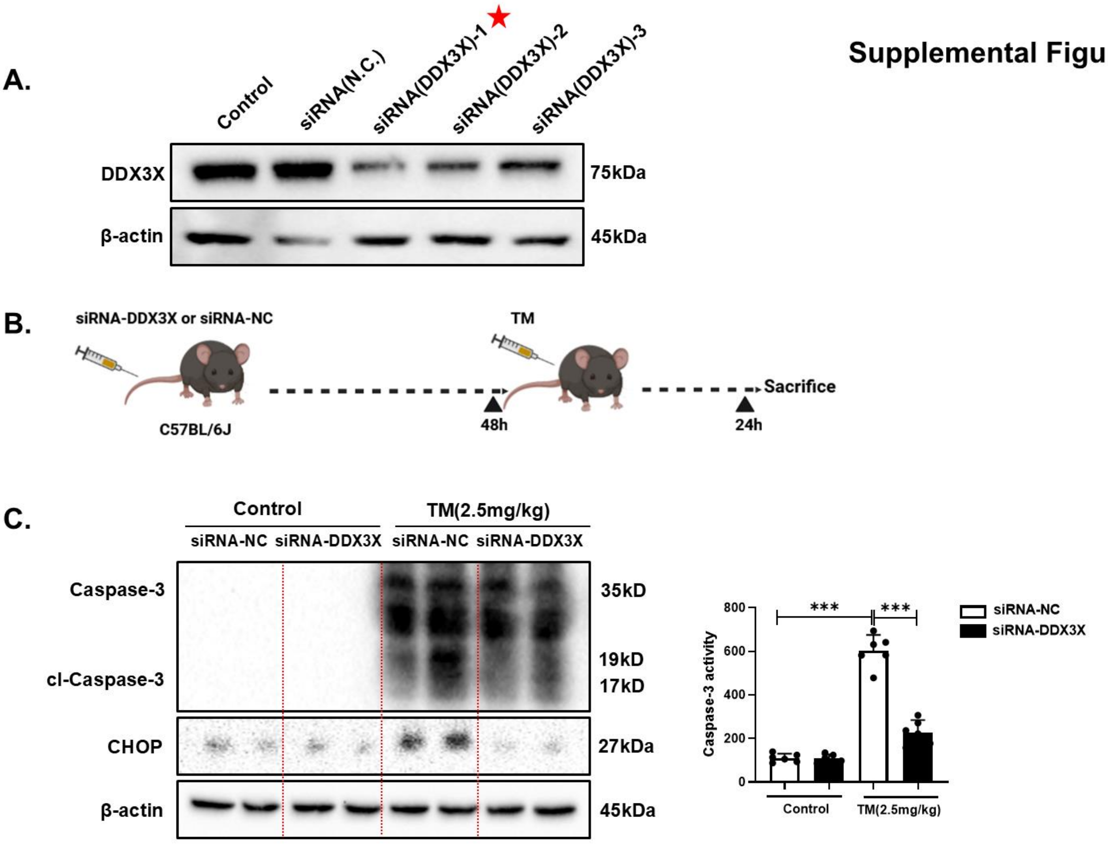

Supplemental Figure 7

A. The efficiency of the DDX3X siRNA was validated. Primary hepatocytes were treated with three DDX3X siRNA oligos for 24 h, and the expression levels of the targets were measured via WB. **B.** C57BL/6J mice were treated with siRNA-NC or siRNA-DDX3X for 48 h and then intraperitoneally injected with TM (2.5 mg/kg) for 24 h. **C**. The caspase activity and expression changes in TM-induced liver injury of mice. *** denotes a p value of <0.001.

Supplemental Figure 8

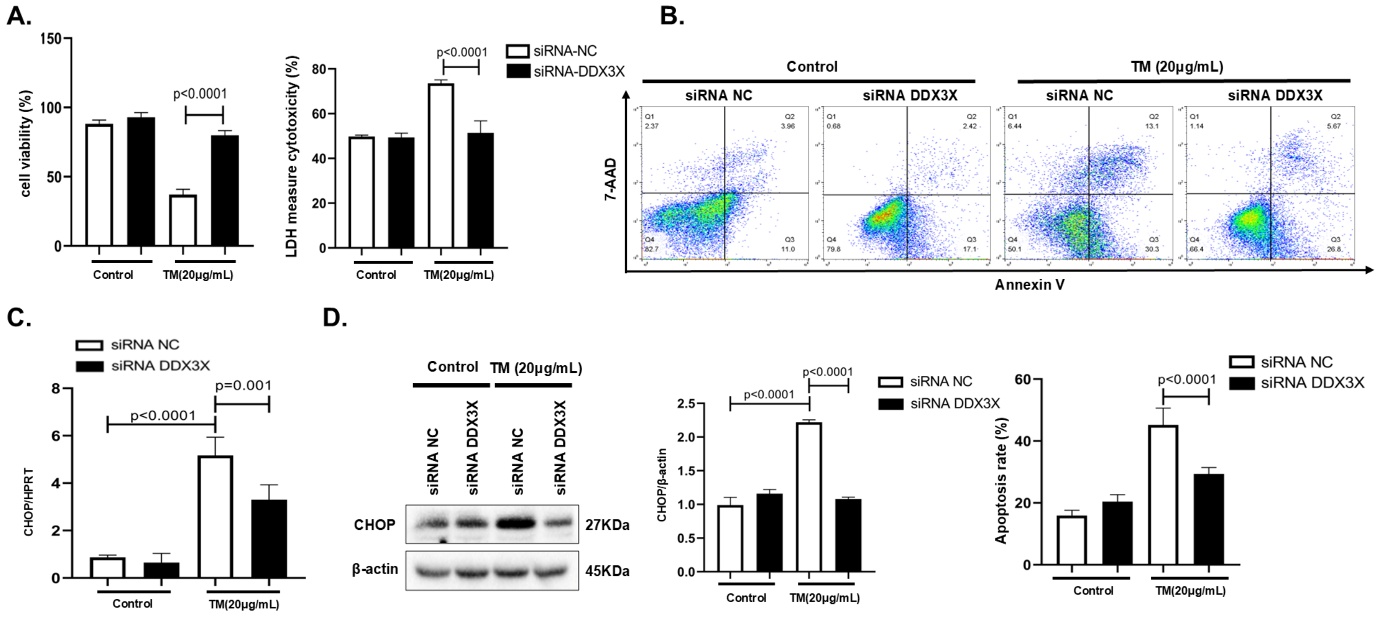

Supplemental Figure 8

Primary hepatocytes were transfected with negative control (NC) siRNA or DDX3X siRNA 24 h before TM treatment. **A.** Cell viability was measured by a CCK-8 assay. Cell death examined by LDH release. **B.** Cell apoptosis was measured by flow cytometry. The different populations of cells were recognized according to their staining: the lower left quadrant was identified as live cells, the lower right quadrant was identified as early apoptotic cells, the upper left quadrant was identified as necrotic cells, and the upper right quadrant was identified as late apoptotic cells. The percentage of apoptotic cells was calculated as the sum of early and late apoptotic cells. **C.** mRNA levels of CHOP were assessed by RT–PCR. **D.** The expression level of CHOP was examined via WB.

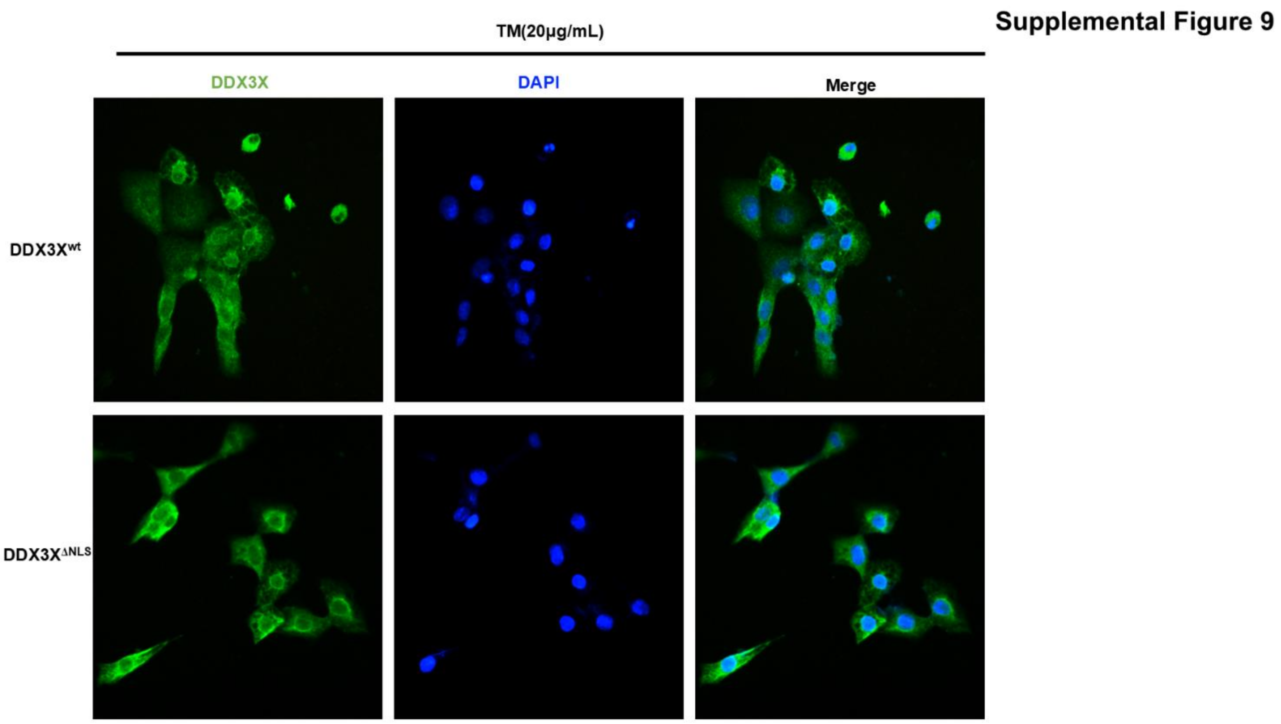

Supplemental Figure 9

The efficiency of the DDX3X$^{\Delta NLS}$ plasmid was validated. Primary hepatocytes were treated with the DDX3X$^{\Delta NLS}$ or DDX3X^{wt} plasmid for 36 h, and the subcellular localization of DDX3X was observed via immunofluorescence staining.

Supplemental Figure 10

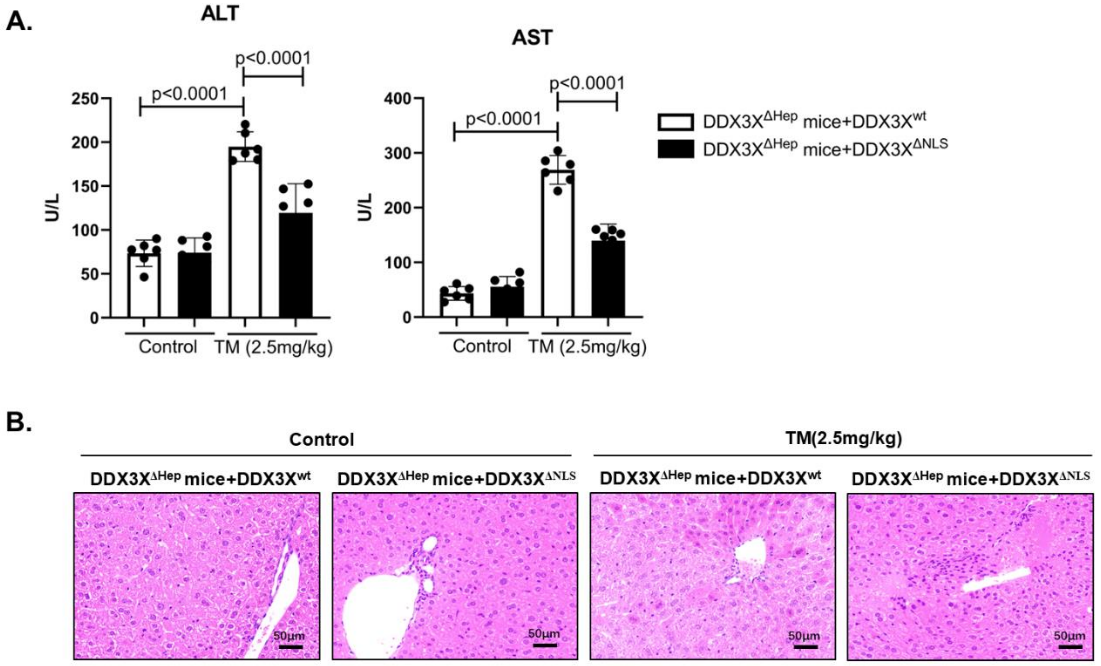

Supplemental Figure 10

The DDX3X^{wt} plasmid (expressing wild-type DDX3X) and DDX3X$^{\Delta NLS}$ plasmid (expressing DDX3X deficient in nuclear localization signals) were transfected in DDX3X$^{\Delta Hep}$ mice, and then intraperitoneally injected with TM (2.5 mg/kg) for 24 h. **A.** Liver injury was assessed by AST and ALT measurements. **B.** Pathological analysis of mouse livers via H&E staining.

Supplemental Figure 11

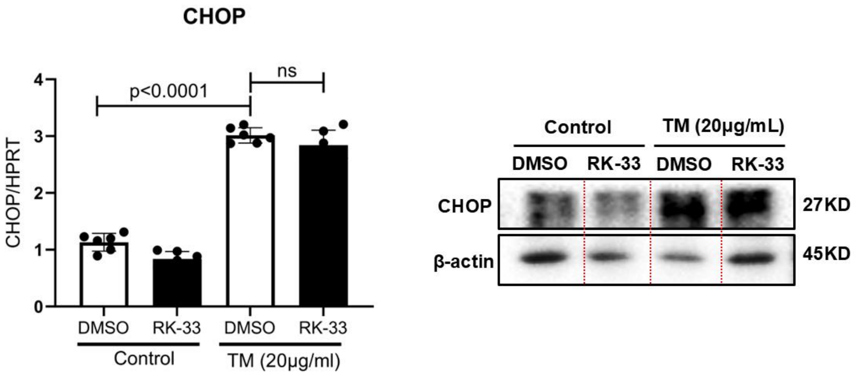

Supplemental Figure 11

The helicase activity of DDX3X were inhibited by RK-33, a small-molecule inhibitor. The primary hepatocytes were treated by RK-33 and were stimulated with TM for 24h, then the expression of CHOP was examined.

Supplemental Figure 12

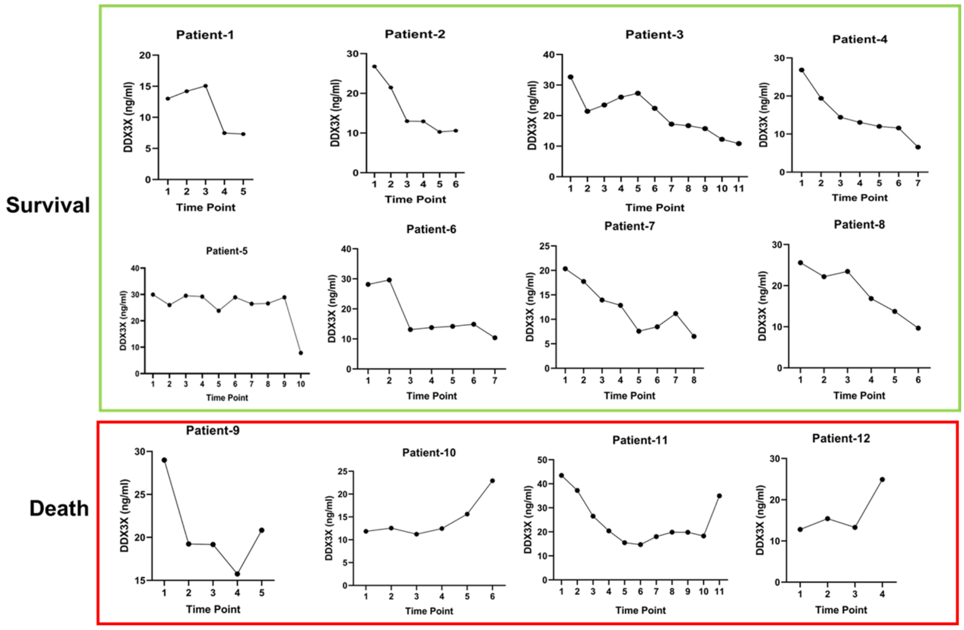

Supplemental Figure 12

Dynamic serum DDX3X levels in 12 patients with LF, including four who died and eight who survived.

Supplemental Figure 13

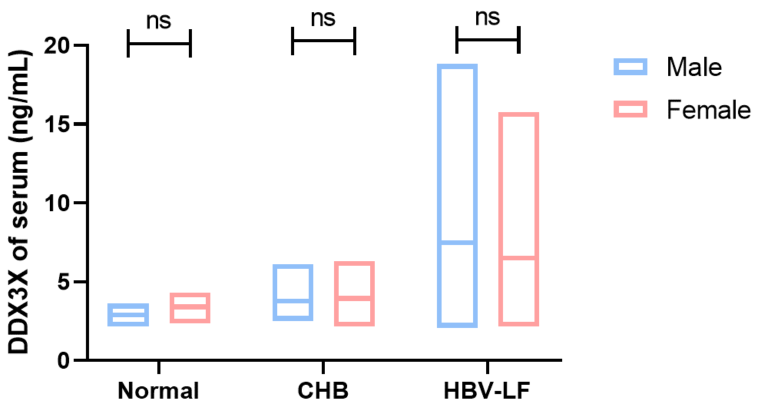

Supplemental Figure 13

The analysis of DDX3X serological levels of gender differences in different cohorts of clinical samples.

Supplemental Tables

Supplemental Table 1

Demographic and clinical characteristics of the different serum study groups

Parameters	Control (n=30)	CHB (n=60)	HBV-ACLF (n=60)	P-value
Age (years)	48±8	40±9	47±13	0.004
Gender (male/female)	19/11	36/24	43/17	
ALT (U/L)	20.3±4.2	40.4±27.5	208.5±321.7	0.007
AST (U/L)	17.6±4.5	37.5±24.5	186.7±222.6	<0.0001
Total bilirubin (μmol/l)	8.0±3.1	19.2±12.4	329.2±169.1	<0.0001
Albumin (g/L)	43.0±3.5	36.1±2.3	31.4±4.1	<0.0001
HBsAg positive (n)	-	60	60	-
HBV DNA (log_{10} copies/mL)	-	4.3±1.2	3.5±1.2	0.0004
Prothrombin time (s)	12.0±0.6	12.4±0.9	26.5±10.6	<0.0001
International normalized ratio	1.0±0.2	1.2±0.1	2.3±1.1	<0.0001

CHB: Chronic hepatitis B; HBV-ACLF: Hepatitis B virus related acute on chronic liver failure; ALT: Alanine aminotransferase; AST: Aspartate aminotransferase; TBIL: Total bilirubin; Alb: Albumin; PT: Prothrombin time; INR: International normalized ratio

Supplemental Table 2

Sequences list of siRNAs

Genes	Sequences
DDX3X-1 sense (5'-3')	CCUGAACUCUUCAGAUAAUTT
DDX3X-1 antisense (5'-3')	AUUAUCUGAAGAGUUCAGGTT
DDX3X-2 sense (5'-3')	CCGCCACACUAUGAUGUUUTT
DDX3X-2 antisense (5'-3')	AAACAUCAUAGUGUGGCGGTT
DDX3X-3 sense (5'-3')	GCAGCAAGAGGACUGGAUATT
DDX3X-2 antisense (5'-3')	UAUCCAGUCCUCUUGCUGCTT

Supplemental Table 3

List of primers

Genes	Primers
Mus-HPRT Forward (5'-3')	AGTCCCAGCGTCGTGATTAG
Mus-HRRT Reverse (5'-3')	GCCTCCCATCTCCTTCATGA
Mus-DDX3X Forward (5'-3')	TGGAGGAAGTACAGCAAGCA
Mus-DDX3X Reverse (5'-3')	TCCCCTTGATCCACTTCCAC
Mus-Grp78 Forward (5'-3')	CTTTGATCAGCGGGTCATGG
Mus-Grp78 Reverse (5'-3')	AGCTCTTCAAATTTGGCCCG
Mus-CHOP-TSS Forward (5'-3')	CCTCATCCCTTACATCCGCC
Mus-CHOP-TSS Reverse (5'-3')	TGAGAGAAGCGGGTGGACTA
Mus-CHOP Forward (5'-3')	TCACTACTCTTGACCCTGCG
Mus-CHOP Reverse (5'-3')	ACTGACCACTCTGTTTCCGT

Supplemental Table 4

List of primary antibodies

Antibodies	Company	Catalog Number	Application
β-actin	Cell Signaling Technology	4970	WB
DDX3X	Abcam	ab196032	WB
GRP78	Cell Signaling Technology	3177	WB
CHOP	Cell Signaling Technology	2895	WB
Histone H3	Cell Signaling Technology	4499	WB
Tubulin	Cell Signaling Technology	2148	WB
p-MLKL	Cell Signaling Technology	37333	WB
p-RIPK3	Cell Signaling Technology	91702	WB
p-RIPK1	Cell Signaling Technology	53286	WB
GPx4	Cell Signaling Technology	59735	WB
Caspase1	Cell Signaling Technology	83383	WB
NLRP3	Cell Signaling Technology	15101	WB
GSDMD	Cell Signaling Technology	39754	WB
PERK	Cell Signaling Technology	3192	WB
ATF6	Cell Signaling Technology	65880	WB
IRE1α	Cell Signaling Technology	3294	WB
Caspase3	Cell Signaling Technology	9662	WB
DDX3X	Abcam	ab271002	IF
