# Supplementary material for: The cytoplasmic-nuclear transport of DDX3X promotes immune-mediated liver injury in mice regulated by endoplasmic reticulum stress
Source: Cell Death Dis. 2024 Sep 30;15(9):702. doi: 10.1038/s41419-024-07076-9 (PMC11442484; doi:10.1038/s41419-024-07076-9)

Figure 1

B.

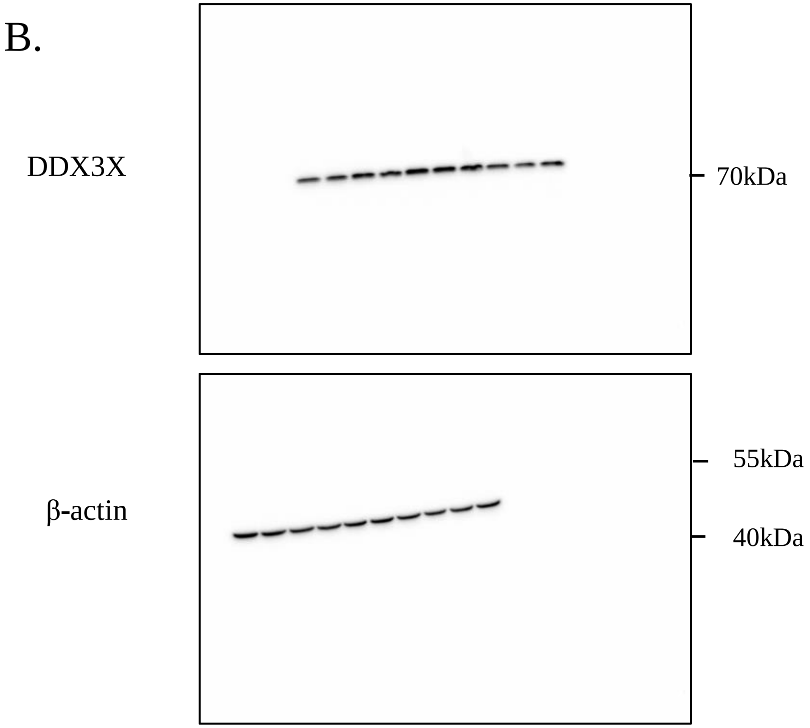

DDX3X
70kDa
β-actin
55kDa
40kDa

E.

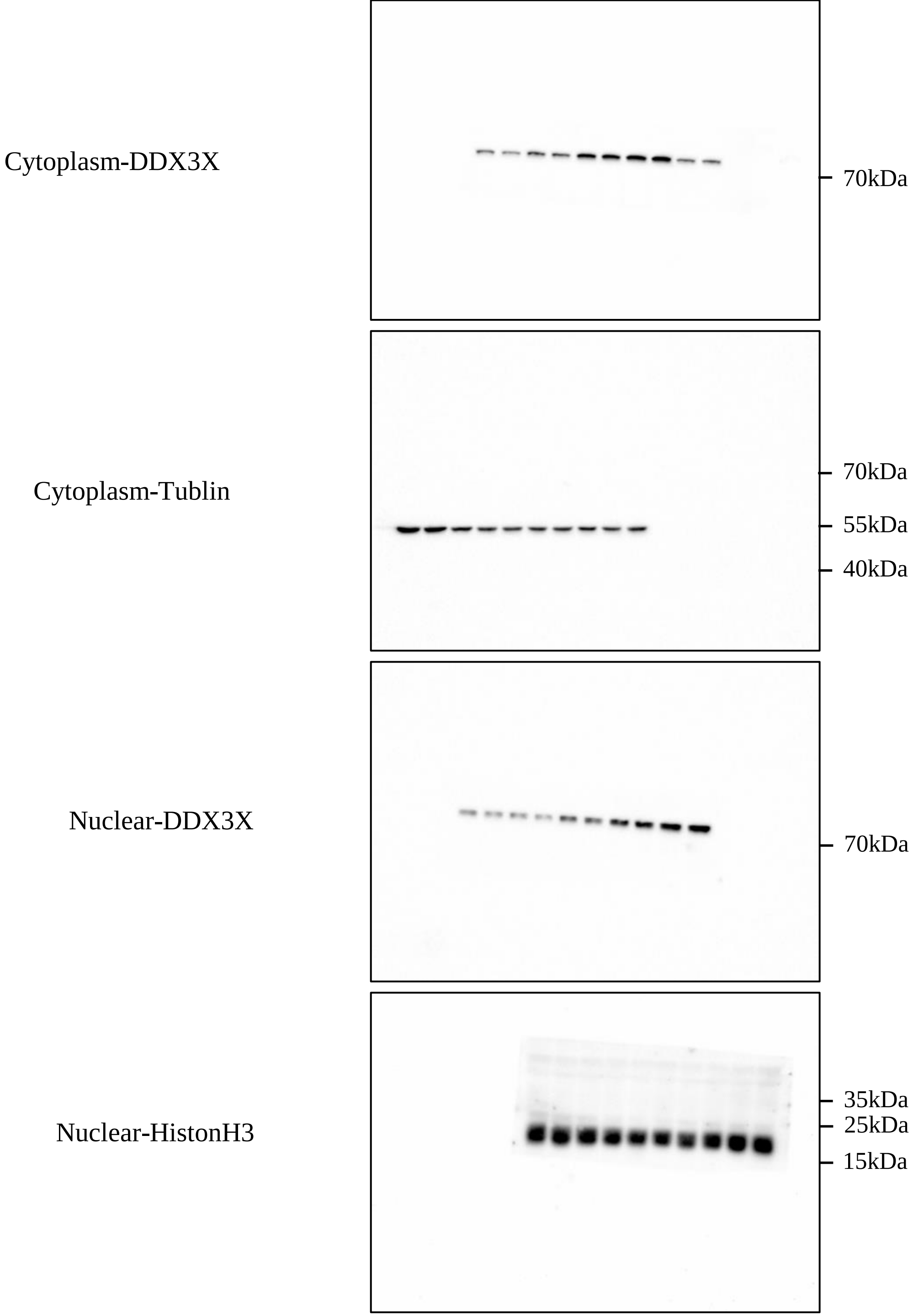

Cytoplasm-DDX3X
70kDa
Cytoplasm-Tublin
70kDa
55kDa
40kDa
Nuclear-DDX3X
70kDa
Nuclear-HistonH3
35kDa
25kDa
15kDa

Figure 2

F.

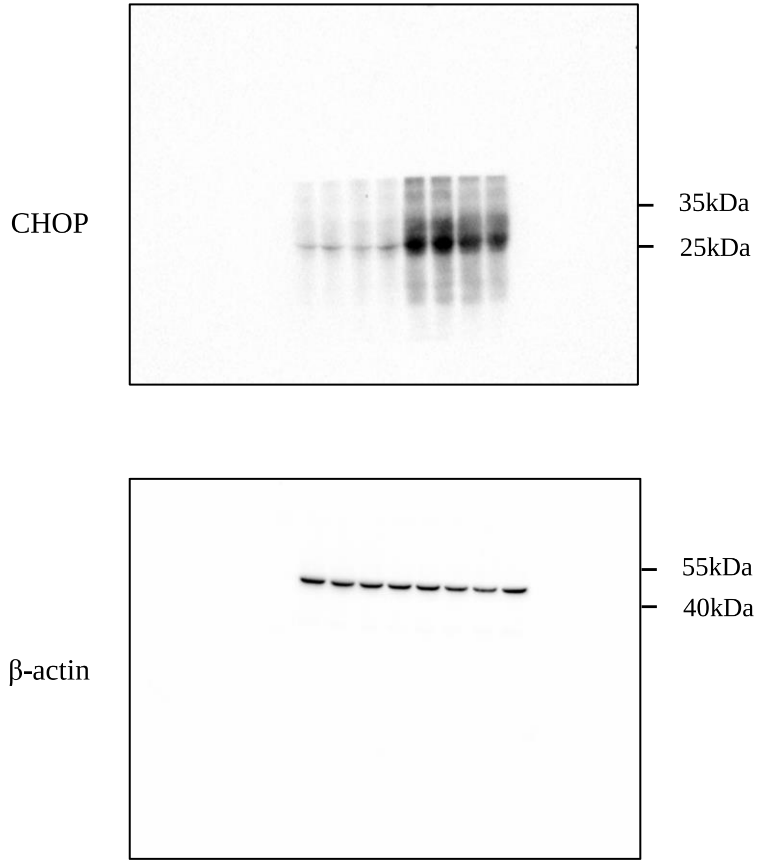

Figure 3

B.

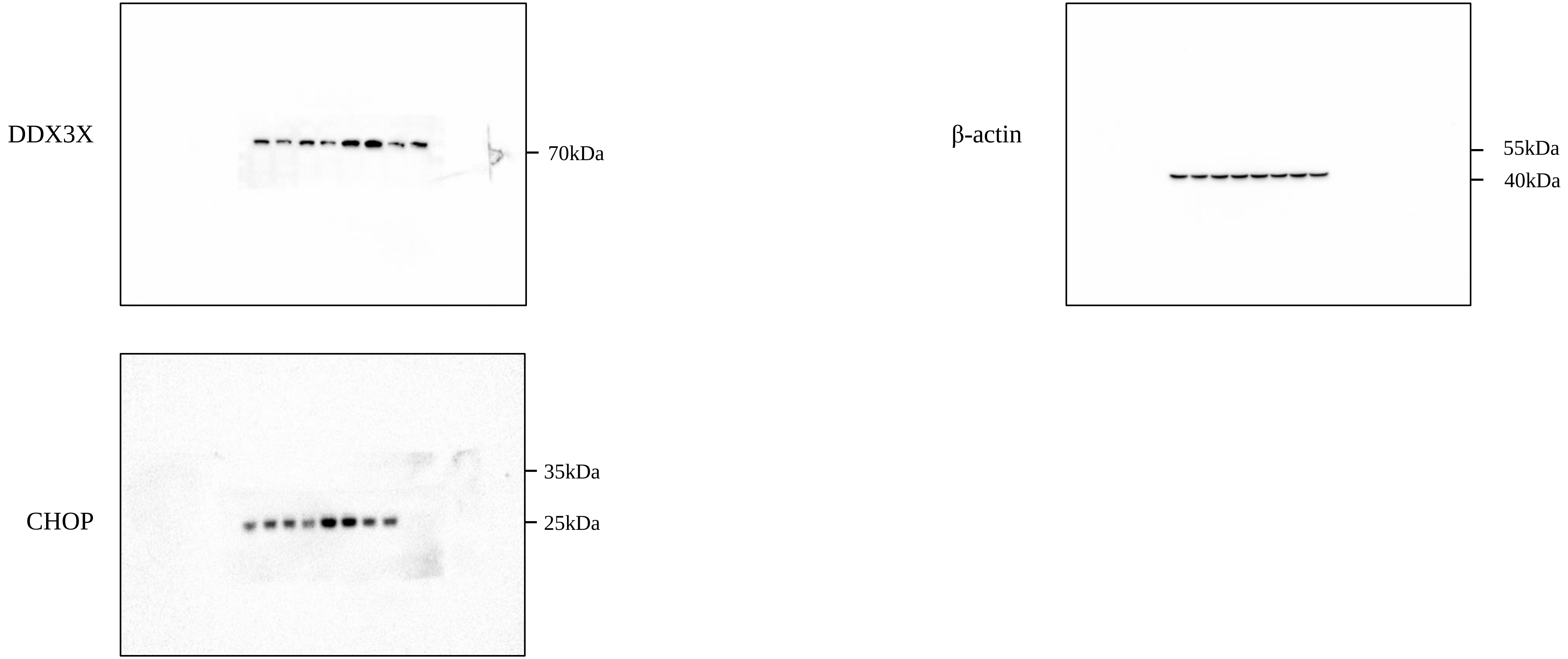

Figure 4

B.

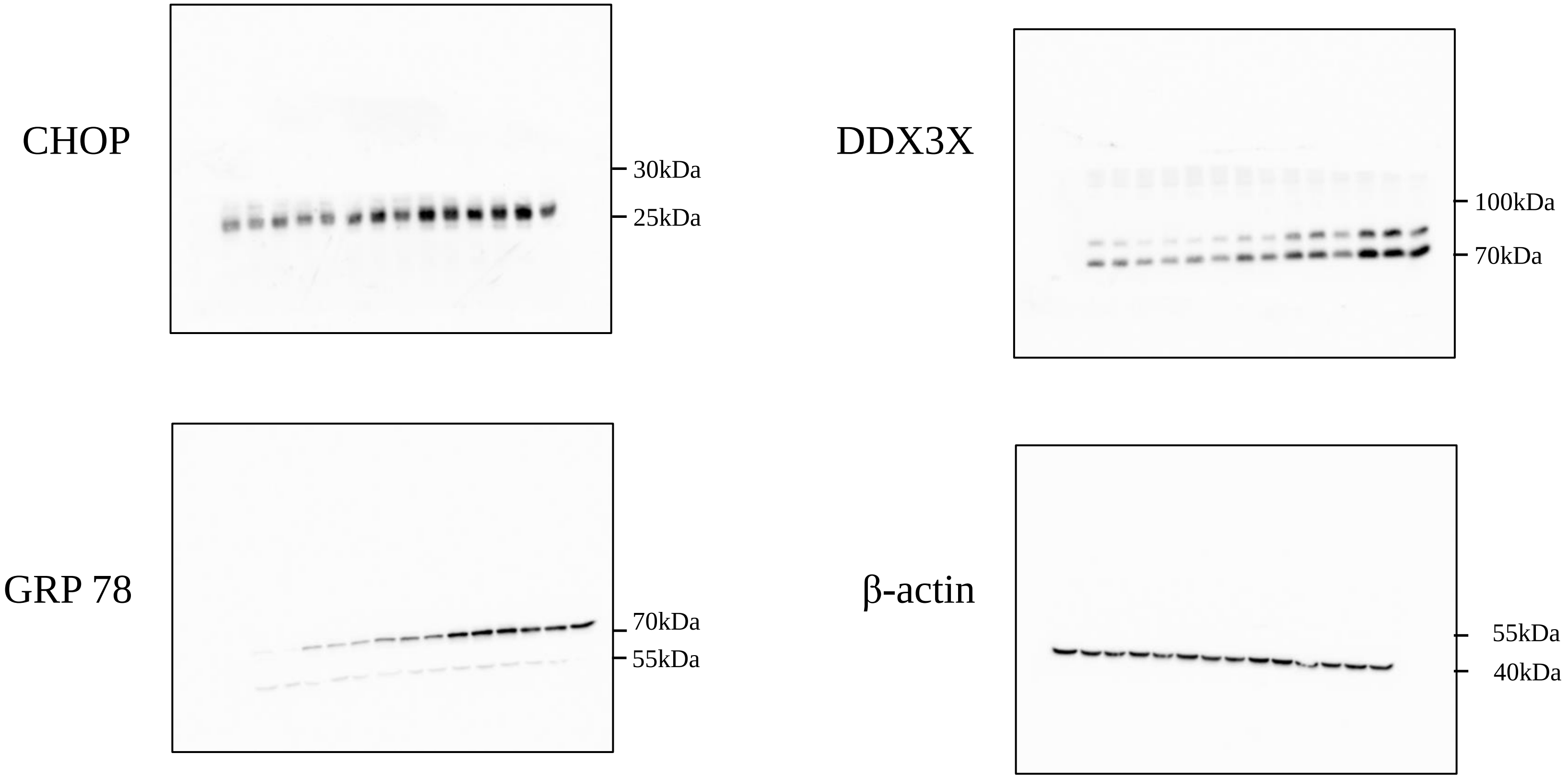
CHOP
30kDa
25kDa
DDX3X
100kDa
70kDa
GRP 78
70kDa
55kDa
β-actin
55kDa
40kDa

Figure 5

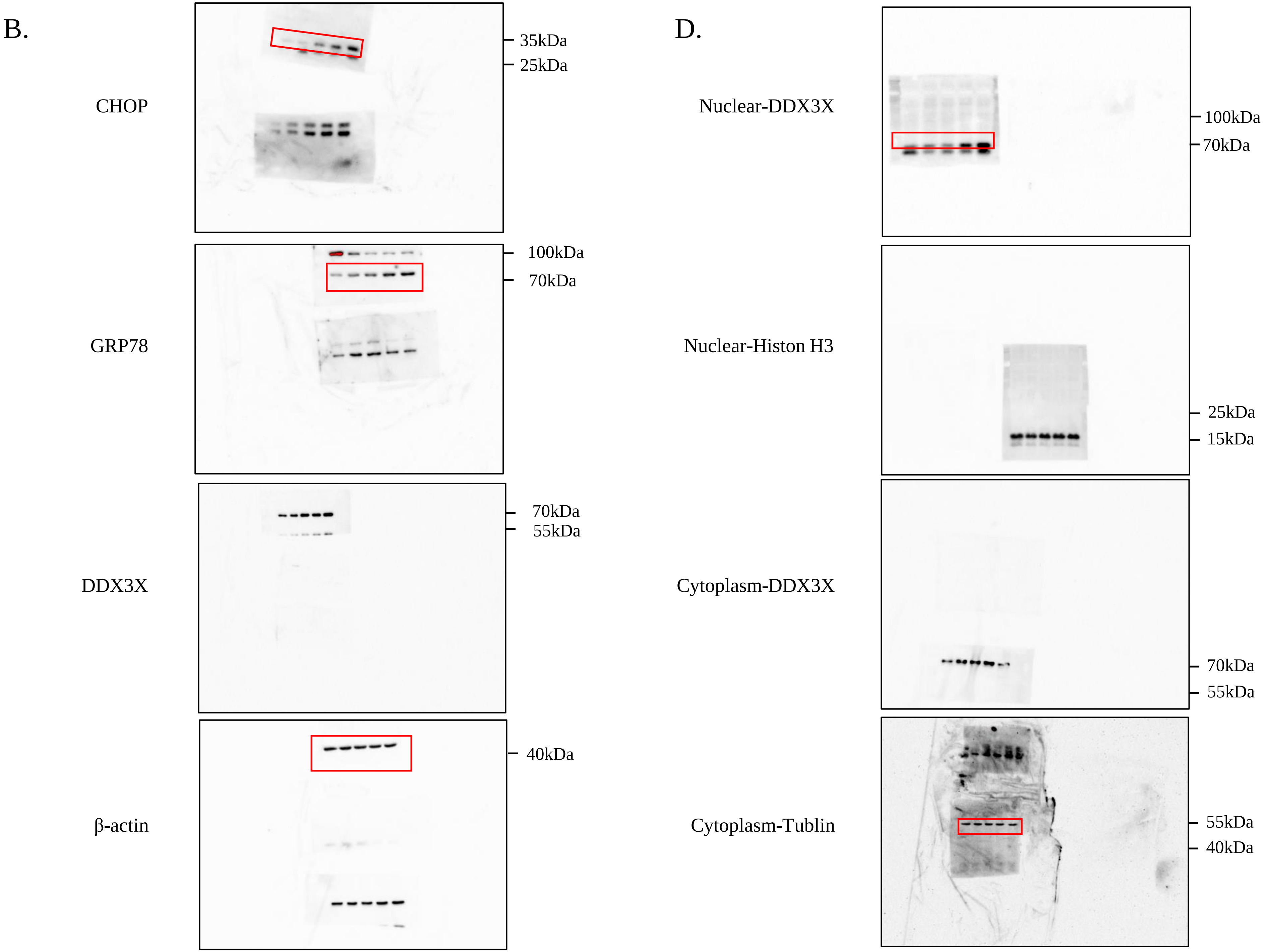
B.
CHOP
35kDa
25kDa
GRP78
100kDa
70kDa
DDX3X
70kDa
55kDa
β-actin
40kDa
D.
Nuclear-DDX3X
100kDa
70kDa
Nuclear-Histon H3
25kDa
15kDa
Cytoplasm-DDX3X
70kDa
55kDa
Cytoplasm-Tublin
55kDa
40kDa

Figure 6

D.

CHOP

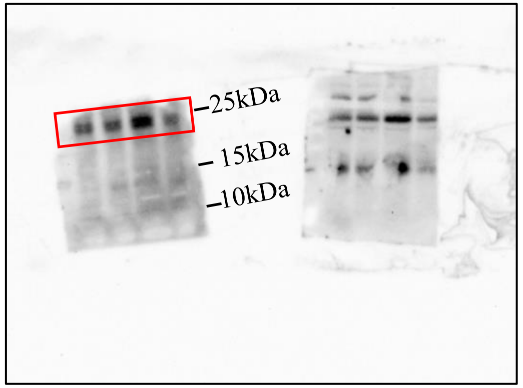

β-actin

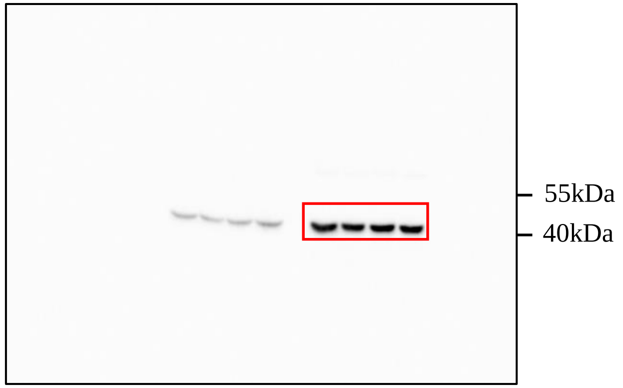

Figure 7

B.

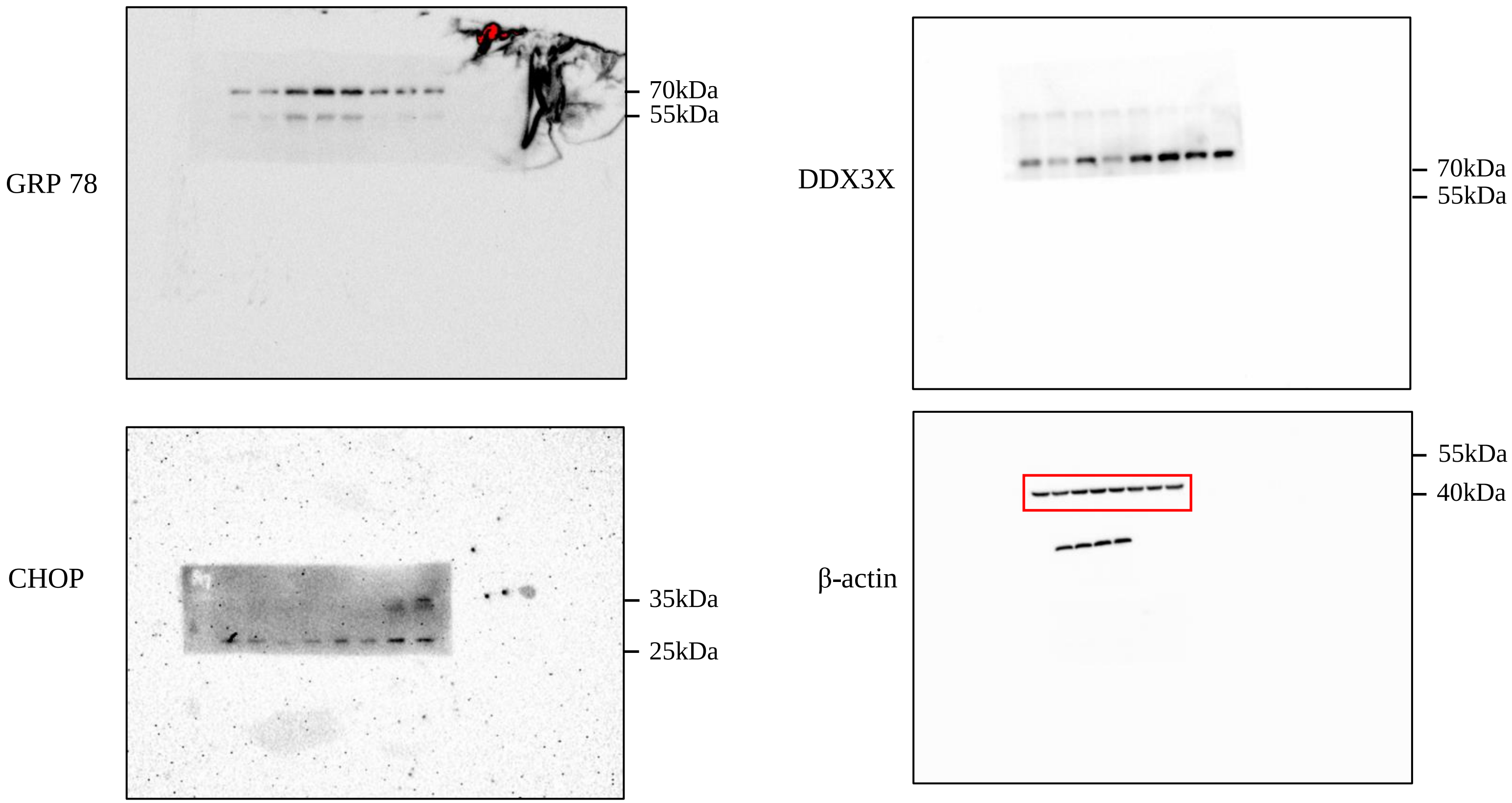
GRP 78
70kDa
55kDa
CHOP
35kDa
25kDa
DDX3X
70kDa
55kDa
β-actin
55kDa
40kDa

F.

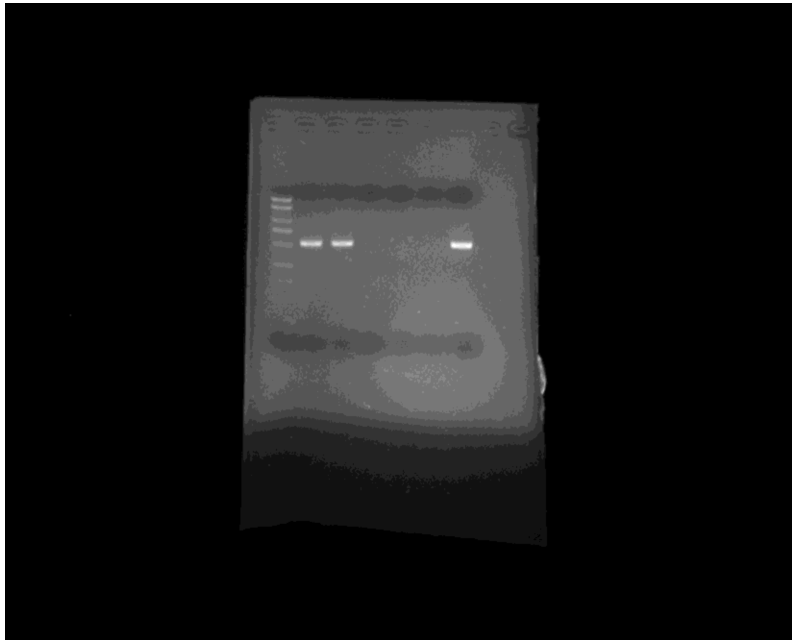

Supplement: Supplementary file 2 — Original western blots [file 41419_2024_7076_MOESM2_ESM.pdf]
